# *In vitro* and *in vivo* safety studies indicate that R15, a synthetic polyarginine peptide, could safely reverse the effects of unfractionated heparin

**DOI:** 10.1002/2211-5463.13240

**Published:** 2021-08-12

**Authors:** Tong Li, Zhiyun Meng, Xiaoxia Zhu, Hui Gan, Ruolan Gu, Zhuona Wu, Taoyun Liu, Peng Han, Jiarui Gao, Su Han, Guifang Dou

**Affiliations:** ^1^ Department of Pharmaceutical Sciences Beijing Institute of Radiation Medicine China

**Keywords:** heparin reversal, heparin substitutes, immunogenicity, polyarginine, protamine

## Abstract

Unfractionated heparin (UFH) is an anionic glycosaminoglycan that is widely used to prevent blood clotting. However, in certain cases, unwanted side effects can require it to be neutralized. Protamine sulfate (PS), a basic peptide rich in arginine, is the only approved antagonist for UFH neutralization. Many adverse reactions occur with the clinical application of PS, including systemic hypotension, pulmonary hypertension, and anaphylaxis. We previously described R15, a linear peptide composed of 15 arginine molecules, as a potential UFH antagonist. In this study, the in‐depth safety of R15 was explored to reveal its merits and associated risks in comparison with PS. *In vitro* safety studies investigated the interactions of R15 with erythrocytes, fibrin, complement, and rat plasma. *In vivo* safety studies explored potential toxicity and immunogenicity of R15 and the UFH–R15 complex. Results showed that both PS and R15 can induce erythrocyte aggregation, thicken fibrin fibers, activate complement, and cause anticoagulation in a concentration‐dependent manner. However, those influences weakened in whole blood or in live animals and were avoided when R15 was in a complex with UFH. We found dramatically enhanced complement activation when there was excess UFH in analyses involving UFH–PS complexes, and a slight increase in those involving UFH–R15 complexes. Within 2 h, R15 was degraded in rat plasma *in vitro*, whereas PS was not. Enhanced creatinine was found after a single intravenous injection of PS or R15 (900 U·kg^−1^, body weight), suggesting possible abnormal renal function. The UFH–PS complex, but not the UFH–R15 complex, exhibited obvious immunogenicity. In conclusion, R15 is nonimmunogenic and potentially safe at a therapeutic dose to reverse the effects of UFH.

## Graphical abstract

Safety studies of R15, a new heparin antagonist, were investigated in comparison with PS (protamine sulfate). R15 was degradable in plasma of rats within 2 h, whereas PS was not. Dramatically enhanced complement activation was observed for UFH–PS complex with excess UFH (unfractionated heparin). UFH–PS complex possessed immunogenicity that was completely absent for the UFH–R15 complex.

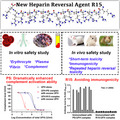

**Abbreviations**
ACT: activated clotting time
AI: aggregation index
ALP: alkaline phosphatase
ALT: alanine aminotransferase
ANOVA: analysis of variance
APTT: activated partial thromboplastin time
AST: aspartate aminotransferase
CPB: cardiopulmonary bypass
CPK: creatine kinase
CREA: creatinine
ELISA: enzyme-linked immunosorbent assay
FDA: Federal Drug Administration
H&E: hematoxylin and eosin
HCT: hematocrit
HGB: hemoglobin
MCH: mean corpuscular hemoglobin
MCHC: mean corpuscular hemoglobin concentration
MCV: mean corpuscular volume
PLT: platelet
PPP: platelet-poor plasma
PS: protamine sulfate
RBC: red blood cell
SEM: scanning electron microscopy
SRBC: sheep red blood cell
UFH: unfractionated heparin
UHRA: universal heparin reversal agents
WBC: white blood cell

Protamine sulfate (PS), a series of cationic arginine‐rich peptides, was discovered in 1868 and approved by the Federal Drug Administration (FDA) for unfractionated heparin (UFH) reversal at the end of extracorporeal circulation in cardiopulmonary surgeries since 1939 [1]. Although PS is widely in clinics used as an UFH antagonist, it has several side effects, including systemic hypotension, anaphylactoid reactions, and catastrophic pulmonary hypertension, and some of these can be life‐threatening [2, 3]. Recently, case reports have described some adverse reactions after PS application, including profound hypotension, cardiac arrest, ventricular fibrillation, bronchospasm with elevated peak airway pressure, decreased oxygen saturation, and catastrophic pulmonary hypertension [4, 5, 6, 7]. PS preparation occurs mainly through extraction from salmon milt, which can result in unstable batches and difficulty in establishing well‐defined quality standards. Furthermore, the supply of protamine depends on marine fish resources, which can be at a shortage risk. Therefore, novel UFH antagonists with an exact known dose–response relationship and minimum side effects are needed not only to meet the needs of the heparin market but also for patients’ safety. The underlying mechanisms of the adverse reactions caused by PS remain unknown by virtue of its complex effects on the blood system, tissues, and organs. The unclear etiology of these side effects can lead to difficulties in the design and screening of novel UFH antagonists. Therefore, researchers often start their studies with the properties of PS that may induce clinical adverse reactions. The properties of PS that may induce adverse reactions are as follows: (a) highly cationic charges that may interact with cells or proteins in the blood system [8, 9]; (b) antigenicity derived from heterogeneous species, which may lead to anaphylaxis [10, 11]; and (c) influences of UFH–PS complex, a polyelectrolyte with a tendency to coacervate and precipitate, on the blood system [12, 13, 14]. Currently, the most advanced clinical heparin antagonist is andexanet alfa (not approved for UFH reversal) [15] or preclinically cationically modified dextrans [16], and universal heparin reversal agents (UHRAs) [17]. Other candidates, including recombinant inactive antithrombin, poly‐l‐lysine, and low‐molecular‐weight protamine, exhibit the ability to reverse UFH in animals [18, 19, 20]. Previously, we reported the potential UFH antagonist R15 (a linear peptide composed of 15 arginine) that has similar efficacy and avoids immunogenicity in comparison with PS [21]. In this article, the factors mentioned above, as well as the efficacy–toxicity relationship, were considered to design the experiments. We aimed to compare the advantages and disadvantages of PS and R15 and to provide the basis for further development of arginine‐based heparin‐neutralizing drugs. The unit of U was mainly used as the concentration and dose unit of PS and R15 in our experiments to facilitate the comparison between PS and R15 at the same efficacy. The therapeutic concentration and dose of UFH is 4 U·mL^−1^ and 300 U·kg^−1^, respectively (for details, please see Materials and methods).

In the present study, *in vitro* and *in vivo* safety studies of R15 and UFH–R15 complex were investigated in comparison with PS. The major findings were as follows: (a) Both PS and R15 induced erythrocyte aggregation, accelerated fibrin polymerization, thickened fibrin fibers, activated complement, and disturbed coagulation function of rat plasma in a concentration‐dependent manner. Those changes were weakened in whole blood and live animals and were avoided by UFH–PS complex and UFH–R15 complex; (b) R15 was degradable in plasma of rats within 2 h, whereas PS was not; (c) dramatically enhanced complement activation was found in UFH–PS complex with excess UFH; (d) 3 times the therapeutic dose of PS and R15 disturbed the function of rat’s kidney. UFH neutralization by PS and R15 at therapeutic dose affected some hematological parameters (RBC, PLT, and HCT) statistically, and those short‐term abnormalities recovered in a week; and (e) UFH–PS complex possessed immunogenicity that was completely avoided by UFH–R15 complex.

## Results

### Potency determination and verification

**Fig. 1:**
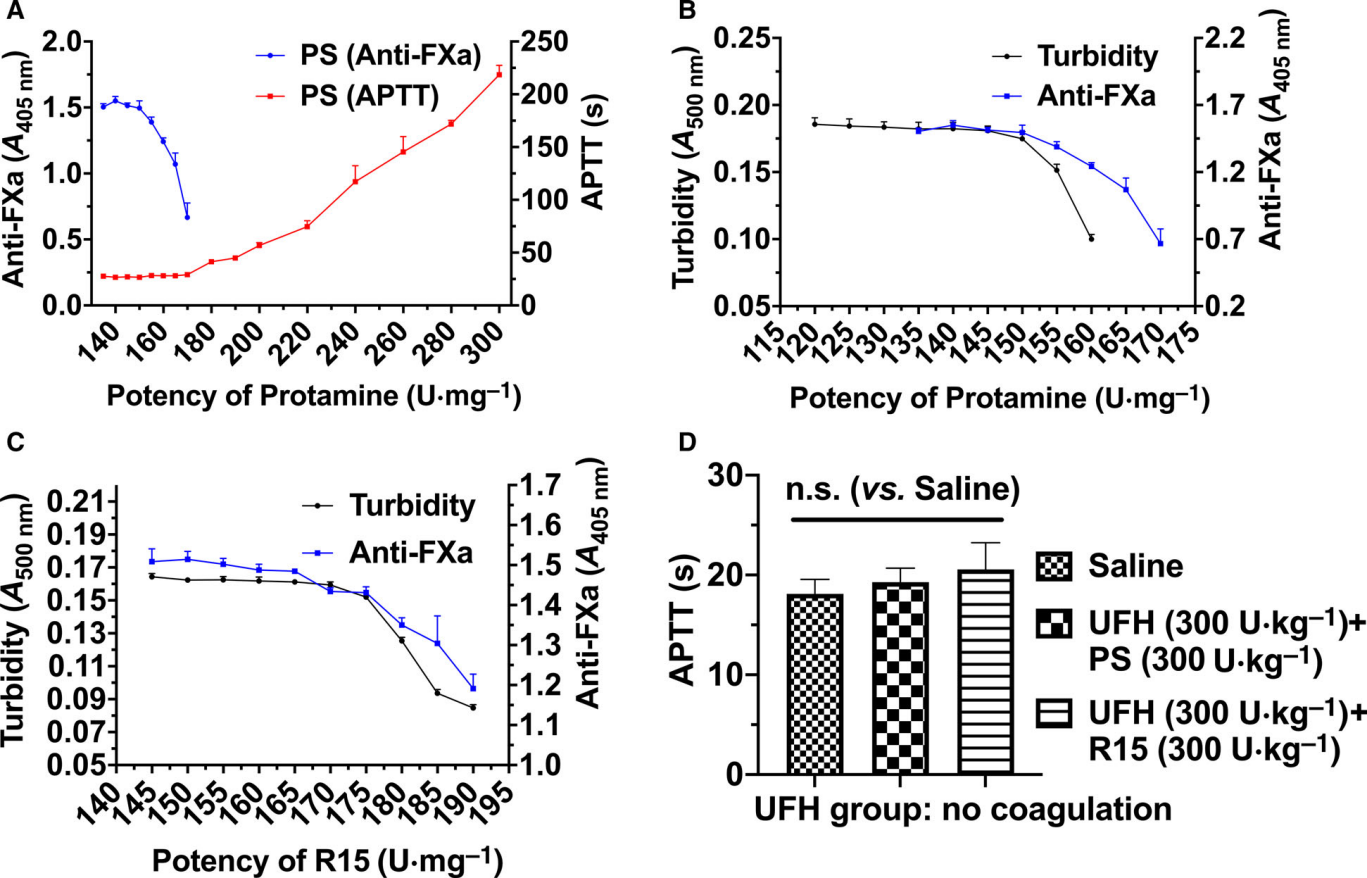
Comparisons of three methods to measure the potency of PS and R15. (A) Comparison of PS potency determination between an active partial thromboplastin time (APTT) clotting assay and anti‐FXa assay. A fixed dose of UFH (APTT: 4 U·mL^−1^, final; anti‐FXa: 1 U·mL^−1^, final) was neutralized by the addition of PS with increasing concentrations. The *y*‐axis on the left represents changes of O.D. value of anti‐FXa assays, and the *y*‐axis on the right represents clotting times measured with APTT assays. (B, C) Potency determination of PS (B) and R15 (C) measured by both turbidity assays and anti‐FXa assays. Comparisons were made between anti‐FXa assays (UFH: 1 U·mL^−1^, final) and turbidity assays (4 U·mL^−1^, final). The x‐axis from (A–C) represents the supposed potency of either PS or R15. (D) APTT measurement of blood samples collected 3 min after UFH neutralization by PS and R15, respectively. The determined potency of PS and R15 was verified in Wistar rats *in vivo* aided by APTT assays (4 groups, *n* = 6 rats per group). Data are presented as mean ± SD. A one‐way ANOVA followed by Dunnett’s multiple comparisons test was used for the statistical analysis. n.s. represents *P* > 0.05. Tests of (A, B, and C) were performed in triplicate.

Three methods were applied to determine the potency of PS and R15 on UFH reversal, and the methods were compared. Anti‐FXa and APTT assays were widely used in efficacy evaluations of PS and PS‐like chemicals [22, 23, 24, 25, 26]. In comparison, determination of turbidimetric change was the most sensitive method compared with the other two methods. As shown in Fig. 1A,B, the potency of PS determined using APTT assays was 170 U·mg^−1^, whereas that using anti‐FXa and turbidity assays was 150 U·mg^−1^. Anti‐FXa and turbidity assays determined the same potency of PS (150 U·mg^−1^) at which the turbidimetric curves reached a plateau with a steeper slope than that of anti‐FXa assays (Fig. 1B). These two methods were used to determine the potency of R15 and found it to be 170 U·mg^−1^ (Fig. 1C). Next, we confirmed the potency via efficacy evaluation in rats *in vivo*. The results from the APTT assays showed that the UFH injected into the rats was fully neutralized by PS and R15 at their determined potency (Fig. 1D).

### Interaction with erythrocytes

We investigated the influences of positively charged PS and R15 on negatively charged erythrocytes. Hemolysis, aggregation, and osmotic resistance of erythrocytes were evaluated.

**Fig. 2:**
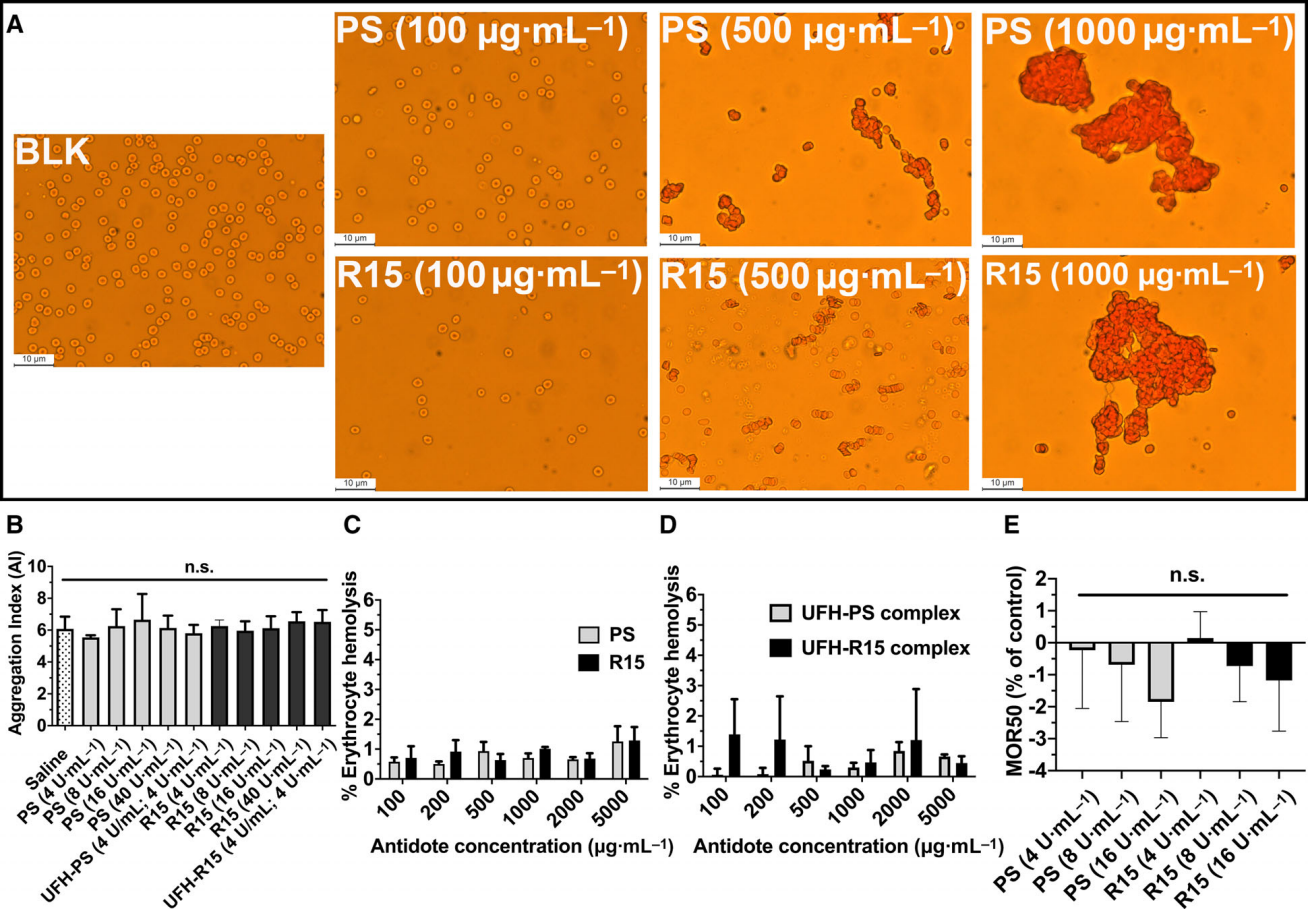
Interaction between PS and R15 on erythrocytes of Wistar rats. (A) Optical microphotographs of erythrocytes incubated with different concentrations of PS and R15 for 1 h at 37 °C, respectively. 50, 100, 500, 1000, and 5000 μg·mL^−1^ of PS and R15 were tested, and only 100, 500, and 1000 μg·mL^−1^ are depicted. BLK represents PBS‐treated erythrocytes taken as the control. There was no sign of aggregation in PS‐ and R15‐treated erythrocytes at concentrations of 50 and 100 μg·mL^−1^. The tendency of erythrocyte aggregation induced by PS and R15 was observed at a concentration of 500 μg·mL^−1^. Bulk aggregates of erythrocytes were seen at concentrations of 1000 and 5000 μg·mL^−1^. All images are at 400× magnification. The scale bar is 10 μm. The whole blood drawn from 3 rats through heart was mixed before the test. (B) The degree of erythrocyte aggregation measured with a viscosity conversion method. Erythrocytes were incubated with PS and R15 at 37 °C for 1 h, followed by the measurement of high and low shear rates. The aggregation index (AI) was calculated from the ratio of the low shear rate (1 s^−1^) to the high shear rate (200 s^−1^). The data are expressed as the mean ± SD, analyzed with a one‐way ANOVA and Dunnett’s multiple comparisons test. The whole blood collected from 6 rats through heart was mixed before the test. (C, D) The degree of hemolysis of erythrocytes incubated with PS, R15 (C), UFH–PS complex, and UFH–R15 complex (D) with varying concentrations for 1 h at 37 °C. PBS and 1% Triton were used as negative (0% of lysis) and positive controls (100% of lysis), respectively. The whole blood collected from 3 rats through heart was mixed before the test. (E) The mean osmotic resistance (MOR50) exposed to PS and R15. Increasing concentrations of NaCl in whole blood were incubated in the presence or absence (control) of different concentrations of PS and R15 at room temperature. MOR50 is the concentration of NaCl at which 50% of erythrocytes were lysed. Saline‐treated samples were set as a control (0%). Erythrocyte suspensions were centrifuged, and the degree of hemolysis was determined from the absorbance of supernatant at 540 nm. The whole blood collected from 3 rats through heart was mixed before the test. The results are expressed as the mean percentage of total hemolysis in comparison with controls ± SD. Kruskal–Wallis test, with Dunn’s multiple comparisons test. n.s. represents *P* > 0.05.

The negative charges on the membranes of erythrocytes were neutralized by positively charged PS, decreasing the electrostatic repulsion force and causing the erythrocytes to aggregate [27]. Under optical observation with a microscope, PBS‐treated erythrocytes were randomly scattered throughout the field of view (Fig. 2, Fig. S1). The first evidence of aggregation occurred in PS‐ and R15‐treated erythrocytes at the concentration of 500 μg·mL^−1^ (Fig. 2A). When the concentration increased to 1000 and 5000 μg·mL^−1^, bulk aggregates of erythrocytes were observed in both PS‐ and R15‐treated erythrocytes. To further investigate the degree of erythrocyte aggregation induced by PS and R15 below 500 μg·mL^−1^, the apparent viscosity of whole blood incubated with the test substances (PS, R15, and UFH–PS and UFH–R15 complexes) at a high and low shear rate was measured using a rheometer, and the aggregation index of erythrocytes was calculated using Eqn (2). No erythrocyte aggregation was found in any tested concentrations from 4 to 40 U·mL^−1^ (40 U·mL^−1^ is 10 times the mean concentration (4 U·mL^−1^) of UFH expected in patients undergoing CPB surgery [28]) (Fig. 2B). In erythrocyte hemolysis assays, up to 5000 μg·mL^−1^, the degree of hemolysis induced by PS, R15, and UFH–PS and UFH–R15 complexes was less than 2% (Fig. 2C,D).

To investigate the influences of PS and R15 on membranes of erythrocytes, erythrocyte osmotic resistance assays were carried out according to the method described elsewhere [16]. An increasing susceptibility to hypotonic lysis was found after incubating erythrocytes with PS and R15 at increasing concentrations, though there was no statistical significance in PS‐ and R15‐treated groups, compared with the vehicle group (Fig. 2E).

### Interaction with fibrin

**Fig. 3:**
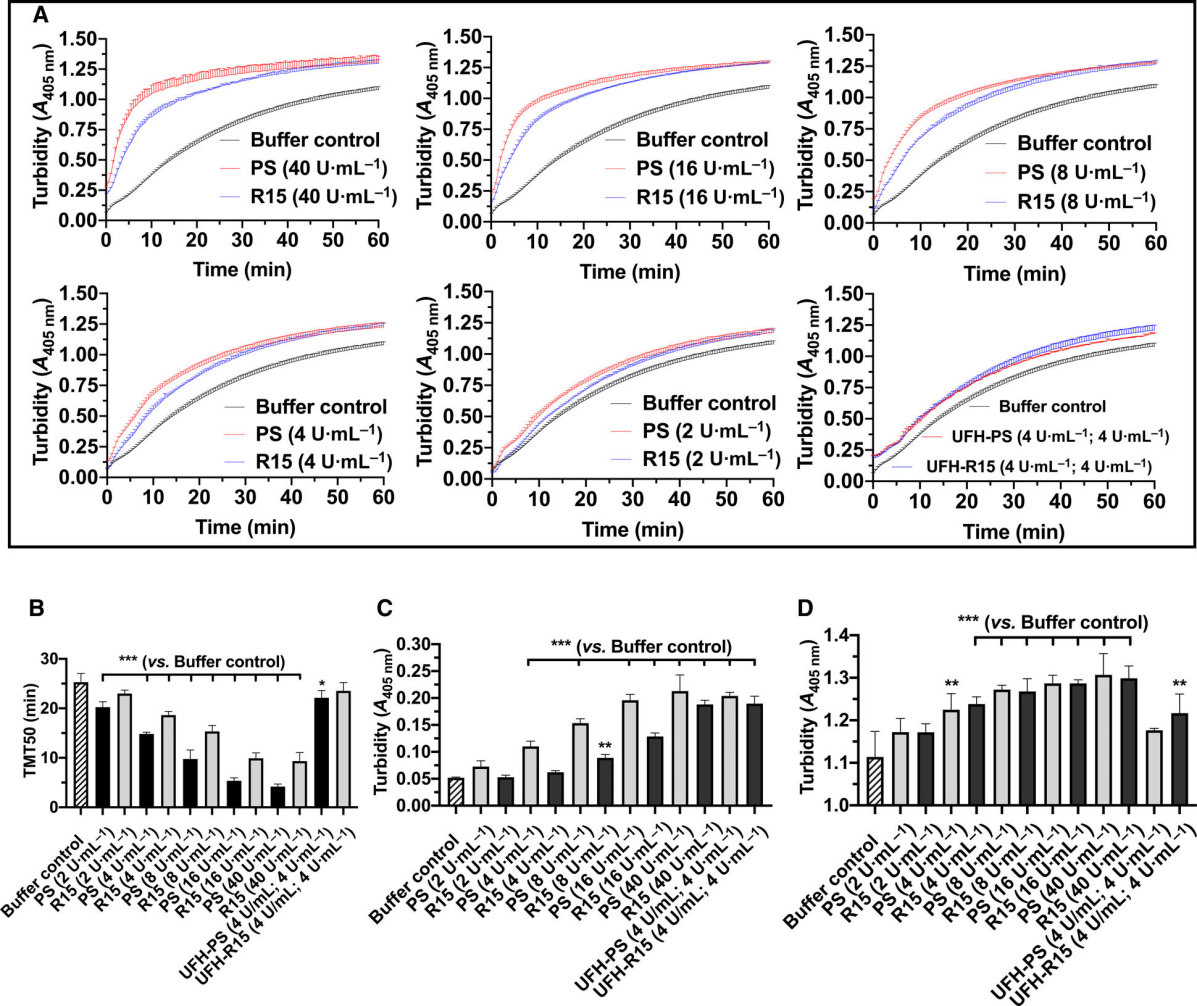
Impact of PS and R15 on pure fibrin formation. (A) Pure fibrin polymerization curves influenced by varying concentrations of PS, R15, UFH–PS complex, and UFH–R15 complex. Fibrinogen incubated with or without (control) PS and R15 for 10 min at 37 °C turned into fibrin after the addition of thrombin and CaCl_2_, resulting in enhanced turbidity at 405 nm. Turbidimetric changes in fibrin polymerization curves were recorded with microplate reader at 405 nm every 30 s for 60 min at 37 °C. (B) Time taken for half maximal turbidity (TMT50) calculated from fibrin polymerization curves. Turbidity was recorded before fibrin polymerization (C) and at the end of fibrin polymerization (D). (B) The addition of PS and R15 into fibrinogen increased the turbidity concentration‐dependently. (D) Turbidity at the end of fibrin polymerization. The data are expressed as the mean ± SD, analyzed with a one‐way ANOVA and Dunnett’s multiple comparisons test. *P < 0.05, **P < 0.01, and ***P < 0.001 vs. buffer control. Tests were performed in triplicate.

We investigated the interaction of PS and R15 with pure fibrinogen using a modified turbidimetric fibrin polymerization assay [24]. Before the initiation of polymerization, fibrinogen containing PS and R15 was measured at 405 nm with a microplate reader. The mixing of PS and R15 with fibrinogen increased the O.D. at 405 nm, which may be ascribed to negatively charged fibrinogen binding to positively charged PS and R15, as shown in Fig. 3C.

When the polymerization was initiated, a turbidimetric curve with slope was produced by recording the absorbance at 405 nm. The curve became steeper when higher concentrations of PS and R15 were added (Fig. 3A). The curves were fitted using nonlinear regression (four parameters), and the time taken for TMT50 was achieved. The addition of PS and R15 shortened the TMT50 in a concentration‐dependent manner, indicating a faster fibrin polymerization when increased concentrations of PS and R15 were added (Fig. 3B). At the end of the polymerization, elevated turbidities were observed in each group compared with the buffer controls, indicating that the changes in the polymerized fibrin fibers were influenced by PS and R15 (Fig. 3D). For fibrin treated with UFH–PS and UFH–R15 complexes, the slight increase in turbidity before the thrombin addition or at the end of the polymerization may be ascribed to their intrinsic turbidity.

**Fig. 4:**
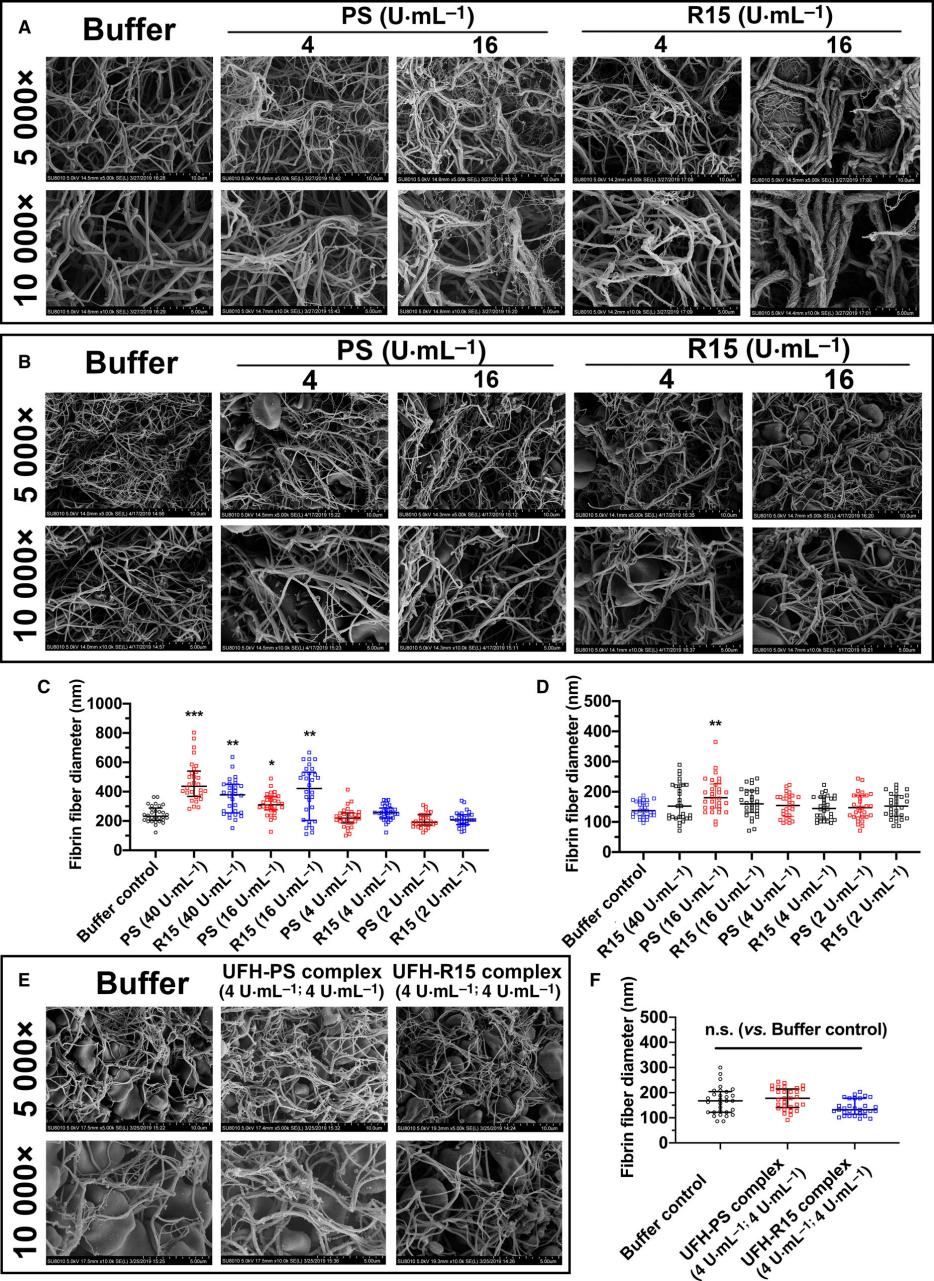
Morphology of fibrin strands formed in fibrinogen and whole blood of Wistar rats in the presence of PS, R15, and UFH–PS and UFH–R15 complexes with varying concentrations. The clot samples, whether pure fibrin fibers or whole blood fibrin fibers, were fixed with 2.5% glutaraldehyde for SEM observation. Images of all clots were captured from different areas at 5000× and 10 000× magnifications. The fiber diameters of 30 fibrin fibers from four separate areas of each image (prepared in a blinded fashion) were measured with imagej. (A) Characteristics of pure fibrin formed by adding thrombin and CaCl_2_ into fibrinogen in the presence of PS and R15 with increasing concentrations. PS and R15 at concentrations of 2, 4, 16, and 40 U·mL^−1^ were tested, and only 4 and 16 U·mL^−1^ are depicted. BLK represents HEPEs buffer‐treated fibrin taken as control. Both PS and R15 at a concentration of 16 U·mL^−1^ or greater thickened the fibrin fibers and twisted fibrin strands in an irregular manner, which was not found at a concentration of 4 U·mL^−1^ or lower. (B and E) Characteristics of whole blood fibrin of Wistar rats formed in blood in the presence of PS, R15 (B), UFH‐PS complex, and UFH–R15 complex (E). BLK represents HEPE buffer‐treated whole blood taken as control. (B) PS and R15 at concentrations of 2, 4, 16, and 40 U·mL^−1^ were tested, and only 4 and 16 U·mL^−1^ are depicted. (C) Fibrin fiber diameters measured from pure fibrin fibers in the presence of PS and R15. (D, F) Fibrin fiber diameters measured from whole blood clots in the presence of PS, R15 (D), UFH–PS complex, and UFH–R15 complex (F). No obvious changes in fibrin morphology or fibrin fiber diameters were found in the whole blood clots, except for PS‐treated fibrin at a concentration of 16 U·mL^−1^. In test (E), the whole blood was drawn from one rat through heart before the test. The scale bar of (A, B, E) is 10 μm (5000× magnification) and 5 μm (10 000× magnification), respectively. Data are presented as mean ± SD. Comparisons were made using Kruskal–Wallis test, with Dunn’s multiple comparisons test. *P < 0.05, **P < 0.01, and ***P < 0.001 vs. buffer control. n.s. represents *P* > 0.05.

To further investigate the changes in fibrin due to PS and R15, SEM was used to detect fibrin fibers in pure fibrin and whole blood clots. Directed visualization of the fibrin clot structure revealed changes in the morphology and mean fiber diameter of the PS‐ and R15‐treated groups. Compared with the buffer controls, thicker fibrin fibers and disarrayed fibrin strands were observed after incubating fibrinogen with PS and R15. The fibrin fibers twisted around each other, like hemp rope forming an irregular fibrin mesh as opposed to the silk‐like fibrin fibers of the control group. When the concentration of PS and R15 decreased, these changes became less obvious (Fig. 4A, Fig. S2).

The influence of PS and R15 on fibrin in whole blood clots was also investigated. Whole blood drawn from the heart of Wistar rats was added to silicone‐coated tubes containing test substances (PS, R15, and UFH–PS and UFH–R15 complexes). Whole blood treated with 40 U·mL^−1^ of PS did not coagulate over 2 h at 37 °C. The changes seen in pure fibrin (thicker and hemp rope‐liked fibrin fibers) were less obvious in all tested whole blood clots (Fig. 4B,E, Fig. S3).

To further investigate the changes in fibrin fibers due to PS and R15, the mean fiber diameter was measured using imagej software. As seen in Fig. 4C, fibrin fibers treated with PS and R15 at concentrations of 16 and 40 U·mL^−1^, respectively, were statistically thicker than buffer‐treated fibrin. The fibrin diameters increased from approximately 200 nm to approximately 400 nm, leading to the elevated final turbidity observed in the fibrin polymerization assays (Fig. 3D). However, the mean fiber diameter calculated from whole blood clots treated with PS, R15, and UFH–PS and R15 complexes was not statistically significantly different except for PS at 16 U·mL^−1^ (Fig. 4D,F).

### Complement activation assay

**Fig. 5:**
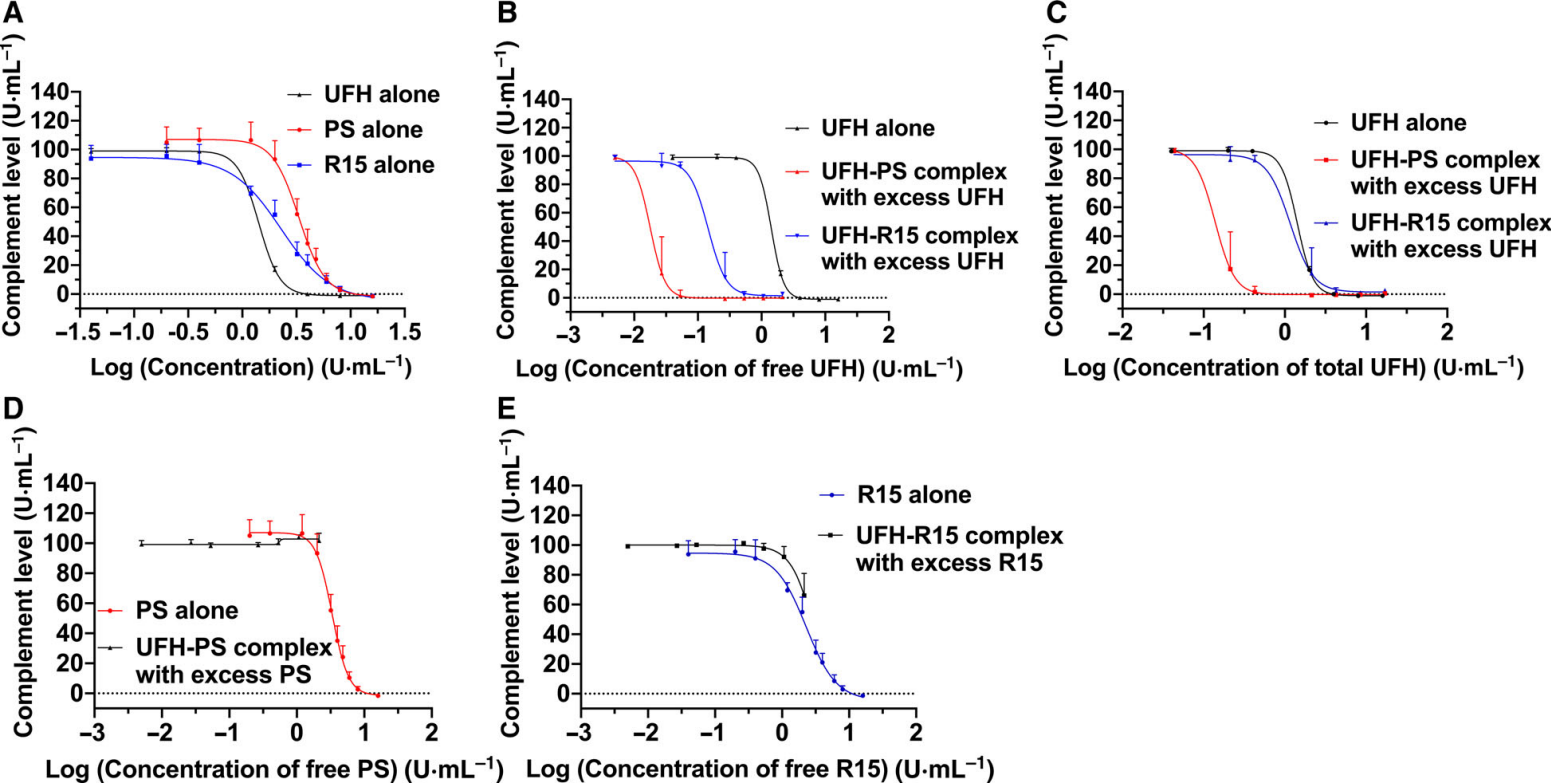
Complement activation by PS, R15, and their UFH binding complexes. The complement activation assay was conducted with a hemolytic complement assay using sera of guinea pigs. The *x*‐axis represents the concentration of test substances in the form of a logarithm. The *y*‐axis represents the complement levels (%). Free UFH was theoretical unbound UFH when UFH was not fully neutralized by PS or R15. Free PS or free R15 was theoretical unbound PS or R15 when UFH was neutralized by overdosed PS or R15. Total UFH was the total amount of UFH, regardless of it being a binding antagonist. For a detailed description of this assay, please see Materials and methods. (A) Influence of UFH, PS, and R15 alone with varying concentrations of complement in the sera of guinea pigs. (B, C) Influence of UFH‐antagonist complex with excess UFH on complement in the sera of guinea pigs. Concentrations of free UFH (B) and total UFH (C) in the form of a logarithm are shown on x‐axis. (D, E) Influence of UFH‐antagonist complex with excess antagonist on complement in the sera of guinea pigs. Concentrations of free PS (D) and free R15 (E) in the form of a logarithm are shown on the x‐axis. Data are presented as mean ± SD. Tests were performed in triplicate.

The complement activation ability of PS, R15, and UFH–PS and UFH–R15 complexes was evaluated by hemolytic complement assay. The results showed that UFH, PS, and R15, when used alone, activated complement in a concentration‐dependent manner (Fig. 5A). The CH50 of UFH, PS, and R15 was 1.42, 3.34 and 2.28 U·mL^−1^, respectively. UFH was the strongest complement activator of the three test substances, and PS was the weakest.

Although UFH and R15 led to complement activation, formation of the UFH–R15 complex at all tested concentrations prevented complement activation (Table 1). Slight degree of complement activation induced by UFH–PS complex was found at final concentrations of 0.2 and 0.4 U·mL^−1^ (Table 2). However, if there was excess UFH after neutralization, UFH–PS or UFH–R15 complexes with excess UFH activated complement in a concentration‐dependent manner (Tables 1 and 2). We calculated the final concentrations of free UFH (Eqn 4) and total UFH (free plus bounded UFH) (Eqn 5) in sera and presented them on the *X*‐axis of two diagrams with complement levels (%) illustrated on the *Y*‐axis (Fig. 5B,C). CH50 of each curve in the two figures was also calculated using nonlinear regression (four parameters). When the concentration of free UFH was used as the *X*‐axis, the CH50 of the UFH–PS complex and UFH–R15 complex was 0.018 and 0.14 U·mL^−1^, respectively. When the concentration of total UFH was used as the *X*‐axis, the CH50 of UFH–PS complex and UFH–R15 complex were 0.14 and 1.16 U·mL^−1^, respectively. If there was excess PS or R15 after neutralization, the mixture of UFH–PS complex with excess PS did not activate complement in any tested concentrations (Fig. 5D). The mixture of UFH–R15 complex with excess R15 activated complement at the maximum tested concentration, maybe because the overdosed R15 activated complement (Fig. 5E).

**Table 1:** Complement level after incubating sera with UFH–R15 complex at different mixing ratios.

Mixing ratio of UFH to R15 before adding into sera (μL·μL^−1^)			Complement level (%)						
		Prepared concentration (UFH and R15)	96 U·mL^−1^	48 U·mL^−1^	24 U·mL^−1^	12 U·mL^−1^	2.4 U·mL^−1^	1.2 U·mL^−1^	0.24 U·mL^−1^
140	160		66.1 ± 14.9	92.0 ± 6.9	97.6 ± 3.5	101.4 ± 0.9	100.2 ± 0.9	99.6 ± 1.4	99.3 ± 0.8
150	150		101.3 ± 1.5	102.2 ± 1.5	100.1 ± 1.2	99.6 ± 2.1	98.7 ± 0.9	98.2 ± 2.1	99.8 ± 0.8
160	140		2.8 ± 0.7	1.2 ± 0.1	2.2 ± 2.3	14.4 ± 17.7	92.5 ± 3.3	92.8 ± 9.2	99.1 ± 1.5
150	0^a^, ^b^		−1.1 ± 0.2	−1.1 ± 0.2	0.1 ± 0.4	16.9 ± 2.2	98.9 ± 1.3	99.2 ± 2.1	98.7 ± 1.9

^a^ Replace with 150 μL buffer solution.

^b^ Assays from Tables 1 and 2 were conducted simultaneously. For convenience of comparison, the complement levels after incubating sole UFH with sera are listed in Tables 2 and 3, respectively.

**Table 2:** Complement levels after incubating sera with UFH–PS complex at different mixing ratios.

Mixing ratio of UFH to PS before adding into sera (μL·μL^−1^)			Complement level (%)						
		Prepared concentration (UFH and PS)	96 U·mL^−1^	48 U·mL^−1^	24 U·mL^−1^	12 U·mL^−1^	2.4 U·mL^−1^	1.2 U·mL^−1^	0.24 U·mL^−1^
140	160		101.7 ± 5.0	103.9 ± 1.6	100.2 ± 2.8	98.8 ± 1.6	98.4 ± 1.8	100.4 ± 2.0	99.3 ± 2.5
150	150		101.4 ± 3.8	100.8 ± 4.2	98.9 ± 3.1	99.1 ± 1.2	74.7 ± 45.5	79.8 ± 18.7	99.1 ± 2.3
160	140		0.6 ± 0.1	−0.3 ± 0.4	−0.5 ± 0.2	−0.7 ± 0.4	1.5 ± 4.0	17.4 ± 25.7	98.9 ± 1.8
150	0^a^		−1.1 ± 0.2	−1.1 ± 0.2	0.1 ± 0.4	16.9 ± 2.2	98.9 ± 1.3	99.2 ± 2.1	98.7 ± 1.9

^a^ Replace with 150 μL buffer solution.

### Influences on coagulation function of rat plasma

**Fig. 6:**
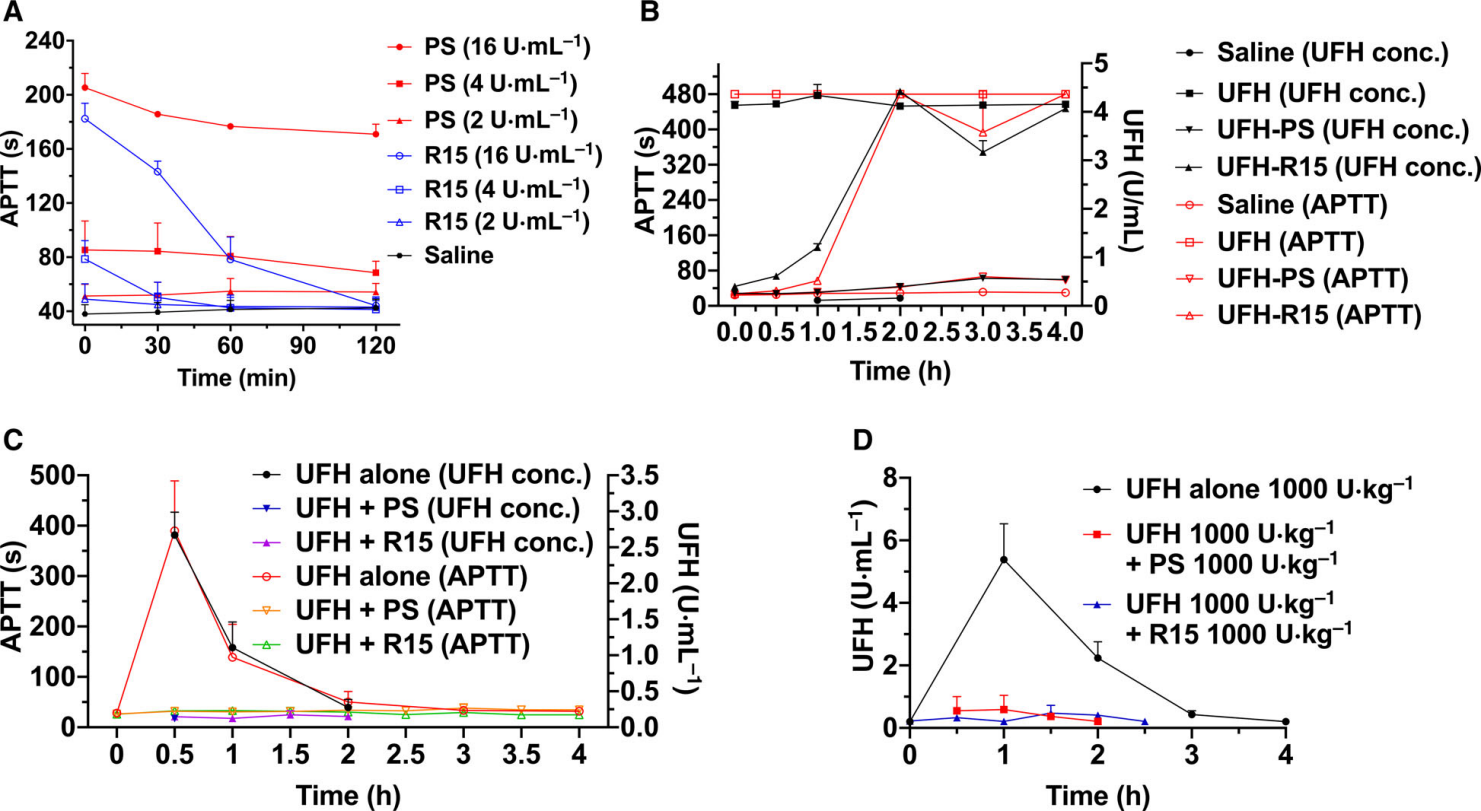
Influence of PS and R15 on plasma of Wistar rats aided by APTT assays and anti‐FXa assays. (A) Incubation of PS and R15 with plasma of Wistar rats *in vitro* for 4 h. PS and R15 elevated APTT of rat plasma concentration‐dependently. The increased APTT induced by R15 declined to normal levels within 2 h, whereas the APTT of PS‐treated plasma was maintained. (B) Incubation of the UFH–PS and UFH–R15 complexes with the plasma of Wistar rats *in vitro* for 4 h. The release of UFH was detected after incubating UFH‐R15 complex with rat plasma aided by APTT assays and anti‐FXa assays. No detectable UFH release was found in the plasma treated with UFH‐PS complex. In test (A and B), the mixed rats’ plasma was prepared from whole blood of 3 rats. (C, D) UFH neutralization by PS and R15 in Wistar rats *in vivo*. Two doses of UFH (C: 300 U·kg^−1^ and D: 1000 U·kg^−1^) were injected into Wistar rats (4 groups, *n* = 6 rats per group), followed by PS or R15 reversal (C: 300 U·kg^−1^ and D: 1000 U·kg^−1^). Both PS and R15 completely neutralized the amount of UFH injected into Wistar rats without detectable UFH release. Data are presented as mean ± SD.

Excess PS increased the value of APTT, perturbing the coagulation of rat plasma [29]. Given the toxicity of PS and R15 that was exhibited in the rat plasma, we investigated the influence of exposure time of PS and R15 to the rat plasma using APTT assays. The presence of PS and R15 elevated the APTT of plasma right after the addition of PS or R15. Then APTT in all R15‐treated plasma declined to the normal level within 2 h, whereas the elevated APTT affected by PS remained (Fig. 6A). Next, we incubated the UFH–PS and UFH–R15 complexes (4 U·mL^−1^; 4 U·mL^−1^) with rat plasma for up to 4 h. The increased UFH levels were detected by both APTT assays and anti‐FXa assays, indicating the disassociation of UFH from UFH–R15 complex (Fig. 6B).

We next performed an *in vivo* experiment with Wistar rats to check whether the UFH release from UFH–R15 complex affects UFH neutralization by R15. Two doses of UFH (300 and 1000 U·kg^−1^) were applied to Wistar rats, followed by corresponding reversal doses of R15 (300 and 1000 U·kg^−1^). UFH levels were monitored with both APTT assays and anti‐FXa assays. The results showed no detectable UFH release in heparinized rats reversed by PS and R15 (Fig. 6C,D).

### Short‐term toxicity in rats

To reveal the toxicity of R15 and UFH–R15 complex, 900 U·kg^−1^ of PS and R15 was intravenously injected into heparinized or nonheparinized Wistar rats, respectively. The blood parameters, biochemical parameters, complement level, coagulation function, and organ toxicity were investigated. The rats that were only injected with saline functioned as the control group.

The results showed that all the test substances did not affect complement levels (Fig. S4A), coagulation function (Fig. S4B), and organs (heart, liver, kidney, spleen, and lung; Figs S5–S9), as well as changes in the lung alveolar areas (Fig. S10). A single injection of PS and R15 alone increased the level of CREA, suggesting potential damages in renal function (Table 3). Compared with the rats in the saline group, the rats treated with UFH or UFH followed by R15 reversal had increased WBC count (Table 4). The injection of R15 alone decreased platelet counts significantly, which still needs further verification (*P* = 0.0465).

**Table 3:** Biochemical parameters analyzed at 1 h after IV single injection of 900 U·kg^−1^ of PS or R15 in heparinized or nonheparinized rats (*n* = 4). Saline‐treated rats functioned as a control group. The statistically significant data are highlighted in bold. Alanine transaminase (ALT); aspartate transaminase (AST); alkaline phosphatase (ALP); creatinine (CREA); creatine kinase (CPK). Data are shown as the median with lower and upper limits. *P < 0.05, **P < 0.01, and ***P < 0.001 vs. saline group. Kruskal–Wallis test, with Dunn’s multiple comparisons test.

Parameters (unit)	Saline	PS 900 U·kg^−1^	R15 900 U·kg^−1^	UFH 900 U·kg^−1^	UFH 900 U·kg^−1^ + PS 900 U·kg^−1^	UFH 900 U·kg^−1^ + R15 900 U·kg^−1^
ALT (U·L^−1^)	67.7 (113.9–1.2)	51.8 (13.1–106.0)	23.8 (50.9–1.0)	43.3 (94.6–7.3)	34.3 (89.8–2.8)	34.5 (116.6–0.7)
AST (U·L^−1^)	22.2 (1.5–44.7)	42.2 (1.1–112.8)	26.2 (0.2–50.8)	19.1 (3.1–33.7)	8.3 (0.2–20.8)	9.8 (0.6–25.0)
ALP (U·L^−1^)	22.1 (17.2–26.2)	37.7 (21.1–51.1)	40.1 (26.4–58.9)	27.1 (14.8–34.7)	28.3 (23.9–34.7)	30.6 (20.6–45.9)
CREA (μmol·L^−1^)	9.4 (0.9–19.5)	119.2* (55.5–**193.3)**	114.4* (32.5–**209.4)**	40.2 (17.7–53.5)	29.2 (3.1–82.7)	42.3 (13.3–74.7)
CPK (U·mL^−1^)	0.228 (0.010–0.487)	0.247 (0.167–0.375)	0.165 (0.003–0.279)	0.124 (0.033–0.219)	0.109 (0.018–0.249)	0.118 (0.077–0.167)

**Table 4:** Blood parameters analyzed at 1 h after an IV single injection of 900 U·kg^−1^ of PS or R15 in heparinized or nonheparinized rats (*n* = 4). Saline‐treated rats functioned as a control group. The statistically significant data are highlighted in bold. White blood cell (WBC); red blood cell (RBC); hemoglobin (HGB); hematocrit (HCT); mean corpuscular volume (MCV); mean corpuscular hemoglobin (MCH); mean corpuscular hemoglobin concentration (MCHC); and platelet (PLT). Data are shown as a median with lower and upper limits. *P < 0.05, **P < 0.01, and ***P < 0.001 *vs*. saline group ^#^ *P* = 0.0465. Kruskal–Wallis test, with Dunn’s multiple comparisons test. The statistically significant data were highlighted in bold.

Parameters (unit)	Saline	PS 900 U·kg^−1^	R15 900 U·kg^−1^	UFH 900 U·kg^−1^	UFH 900 U·kg^−1^ + PS 900 U·kg^−1^	UFH 900 U·kg^−1^ + R15 900 U·kg^−1^
WBC (10^9^ L^−1^)	5.8 (4.1–7.0)	8.7 (6.3–10.3)	7.5 (7.0–8.4)	10.6* (8.5–**12.3)**	6.6 (3.9–9.5)	11.2* (9.6–**15.3)**
RBC (10^12^ L^−1^)	4.27 (4.03–4.62)	4.54 (4.14–5.12)	4.32 (4.18–4.49)	4.03 (3.66–4.47)	4.15 (3.88–4.44)	4.46 (4.31–4.55)
HGB (g·L^−1^)	106.5 (98.0–113.5)	108.4 (104.3–114.7)	106.0 (103.0–110.3)	102.9 (98.2–109.1)	105.6 (103.9–108.0)	109.9 (105.9–111.7)
HCT (%)	25.9 (24.0–28.0)	27.3 (25.1–28.7)	25.6 (25.0–25.9)	24.4 (23.7–25.3)	25.0 (23.4–26.2)	27.1 (25.8–29.1)
MCV (fL)	60.7 (58.4–63.9)	60.4 (56.0–64.7)	59.3 (55.8–62.2)	61.0 (55.8–66.3)	60.6 (55.8–66.9)	60.1 (58.2–62.2)
MCH (pg)	25.9 (24.0–28.0)	27.3 (25.1–28.7)	25.6 (25.0–25.9)	24.4 (23.7–25.3)	25.0 (23.4–26.2)	27.1 (25.8–29.1)
MCHC (g·L^−1^)	411 (401–421)	397 (381–417)	414 (400–430)	422 (405–438)	422 (401–446)	406 (363–429)
PLT (10^9^ L^−1^)	419 (378–492)	440 (482–504)	329*^,#^ (319–**344)**	364 (332–405)	387 (217–471)	377 (364–391)

### Repeated UFH reversal in BALB/c mice

Repeated reversal of UFH by PS and R15 was applied once a week for 5 weeks in BALB/c mice, aided by blood count analysis, antigenicity detection, and histopathological examination. Mice with only saline injection functioned as the control group. No sign of drug‐related toxicity was observed in the tested mice during the study. No statistically significant difference was found in the mean baseline body weights between the randomized treatment groups (Fig. S11). No pathological change was found in the examined organs (heart, liver, spleen, lung, and kidney; Fig. S12–S16 and Table S1), as well as changes in the lung alveolar areas (Fig. S17). No antigenicity was found in any tested group (Fig. S18). Blood counts including WBC, HCT, HGB, MCV, RBC, MCHC, and PLT were carried out to observe changes in blood parameters. In each group (*n* = 16), 6 of 16 had blood counts determined at 1 h after UFH reversal on the 1^st^, 3^rd^, and 5^th^ week (day 1 and days 15 and 29), and the other 10 mice in each group were tested at the 6^th^ week (day 36). Tables 5 and 6 showed increased WBC (found at the 1^st^ and 3^rd^ weeks) and decreased PLT counts (found at the 1^st^ week) in heparinized mice. A slight but significant decline in the RBC count (found at the 3^rd^ week) was observed in PS‐ and R15‐treated heparinized mice, and deceased HCT levels were found in R15‐treated heparinized mice (found at the 3^rd^ week).

**Table 5:** Blood parameters of BALB/c mice treated with UFH alone (300 U·kg^−1^) or followed by PS (300 U·kg^−1^) or R15 (300 U·kg^−1^; *n* = 6). Test substances were given to BALB/c mice once a week for five weeks, and the blood parameters were analyzed at 1 h after administration every other week. Saline‐treated mice were taken as a control group. White blood cell (WBC); red blood cell (RBC); hemoglobin (HGB); hematocrit (HCT); mean corpuscular volume (MCV); mean corpuscular hemoglobin (MCH); mean corpuscular hemoglobin concentration (MCHC); and Platelet (PLT). Data are shown as the median with lower and upper limits. *P < 0.05, **P < 0.01, and ***P < 0.001 *vs*. saline group. Kruskal–Wallis test, with Dunn’s multiple comparisons test. The statistically significant data are highlighted in bold.

Parameters (unit)	Analyzing time	Saline	UFH 300 U·kg^−1^	UFH 300 U·kg^−1^ + PS 300 U·kg^−1^	UFH 300 U·kg^−1^ + R15 300 U·kg^−1^
WBC (10^9^ L^−1^)	1st week	5.0 (3.3–7.2)	10.6*** (7.9–**13.9)**	6.2 (4.9–9.3)	6.5 (6.1–7.1)
	3rd week	4.9 (4.2–5.9)	9.3*** (7.8–**10.8)**	6.8 (6.1–8.9)	6.0 (5.2–6.9)
	5th week	5.6 (4.0–6.9)	6.7 (5.9–7.6)	6.5 (4.8–7.5)	6.8 (3.7–12.2)
HGB (g·L^−1^)	1st week	149.0 (143.6–160.7)	144.1 (134.2–149.9)	145.5 (139.6–150.9)	144.4 (136.1–150.1)
	3rd week	136.0 (133.5–139.1)	129.5 (124.6–137.7)	130.8 (125.7–136.0)	130.4 (122.4–136.3)
	5th week	137.1 (130.2–141.4)	136.9 (131.8–140.9)	139.4 (130.6–147.4)	137.7 (133.7–142.1)
MCH (pg)	1st week	20.7 (20.2–21.3)	20.3 (19.6–21.1)	20.0 (19.6–20.6)	20.6 (19.8–21.1)
	3rd week	20.9 (20.6–21.2)	20.5 (20.3–20.8)	21.1 (20.3–21.5)	21.1 (20.2–21.8)
	5th week	20.3 (19.8–20.5)	20.5 (19.9–21.4)	20.4 (19.5–21.0)	19.9 (19.5–20.2)
MCHC (g·L^−1^)	1st week	398 (388–412)	398 (391–405)	381 (371–394)	402 (385–414)
	3rd week	406 (394–414)	399 (392–404)	407 (395–420)	412 (400–418)
	5th week	387 (380–394)	397* (390–**411)**	394 (385–401)	388 (384–393)
RBC (10^12^ L^−1^)	1st week	7.19 (6.99–7.88)	7.08 (6.56–7.42)	7.27 (6.81–7.67)	7.00 (6.50–7.35)
	3rd week	6.48 (6.39–6.63)	6.30 (6.12–6.71)	6.20* (5.97–**6.41)**	6.17** (6.04–**6.30)**
	5th week	6.74 (6.53–7.04)	6.66 (6.48–6.98)	6.80 (6.62–7.04)	6.90 (6.66–7.06)
MCV (fL)	1st week	52.0 (51.6–52.9)	51.3 (49.7–52.4)	52.5 (51.6–53.0)	51.4 (50.6–52.4)
	3rd week	51.8 (50.9–52.5)	51.5 (50.5–52.4)	51.9 (50.8–52.9)	51.3 (50.4–52.3)
	5th week	52.5 (51.5–53.4)	51.9 (50.8–53.5)	52.1 (50.8–53.2)	51.5 (50.9–52.8)
HCT (%)	1st week	37.4 (36.2–40.8)	36.2 (33.3–37.6)	38.1 (36.0–40.6)	35.9 (32.8–37.8)
	3rd week	33.5 (32.7–34.2)	32.4 (31.4–35.1)	32.2 (30.8–33.9)	31.6* (30.6–**32.8)**
	5th week	35.4 (34.2–36.2)	34.5 (33.2–35.4)	35.4 (33.9–37.3)	35.5 (34.2–37.0)
PLT (10^9^ L^−1^)	1st week	403 (346–432)	357* (334–**378)**	414 (372–450)	403 (360–503)
	3rd week	416 (386–487)	413 (355–449)	419 (379–452)	438 (395–468)
	5th week	479 (405–716)	457 (437–514)	444 (378–482)	447 (405–502)

**Table 6:** Blood parameters of BALB/c mice treated with UFH alone (300 U·kg^−1^) or followed by PS (300 U·kg^−1^) or R15 (300 U·kg^−1^; *n* = 10). Test substances were given to BALB/c mice once a week for 5 weeks, and the blood parameters were analyzed at the 6^th^ week. Saline‐treated mice functioned as a control group. White blood cell (WBC); red blood cell (RBC); hemoglobin (HGB); hematocrit (HCT); Mean corpuscular volume (MCV); mean corpuscular hemoglobin (MCH); mean corpuscular hemoglobin concentration (MCHC); and platelet (PLT). Data are shown as a median with lower and upper limits. *P < 0.05, **P < 0.01, and ***P < 0.001 *vs*. saline group. Kruskal–Wallis test, with Dunn’s multiple comparisons test.

Parameters (unit)	Saline	UFH 300 U·kg^−1^	UFH 300 U·kg^−1^ + PS 300 U·kg^−1^	UFH 300 U·kg^−1^ + R15 300 U·kg^−1^
WBC (10^9^ L^−1^)	4.3 (2.7–6.3)	4.5 (1.6–8.3)	4.2 (2.8–6.8)	4.0 (2.4–6.6)
HGB (g·L^−1^)	154.6 (146.9–160.7)	158.6 (148.4–167.8)	155.1 (144.6–164.7)	153.8 (147.2–165.7)
MCH (pg)	17.7 (16.8–18.4)	17.8 (17.3–18.4)	17.8 (17.1–18.1)	17.5 (16.6–18.2)
MCHC (g·L^−1^)	336 (321–349)	343 (331–360)	341 (330–349)	337 (322–350)
RBC (10^12^ L^−1^)	8.74 (8.18–9.12)	8.89 (8.06–9.41)	8.70 (8.37–9.07)	8.78 (8.46–9.29)
MCV (fL)	52.6 (51.5–54.2)	52.0 (51.0–53.4)	52.3 (50.8–53.9)	52.0 (50.4–53.2)
HCT (%)	45.9 (42.4–47.8)	46.2 (41.2–49.0)	45.2 (41.1–47.7)	45.6 (43.6–47.8)
PLT (10^9^ L^−1^)	436 (375–510)	433 (379–548)	399 (353–458)	417 (386–461)

### Immunogenicity of the UFH–PS and UFH–R15 complexes

**Fig. 7:**
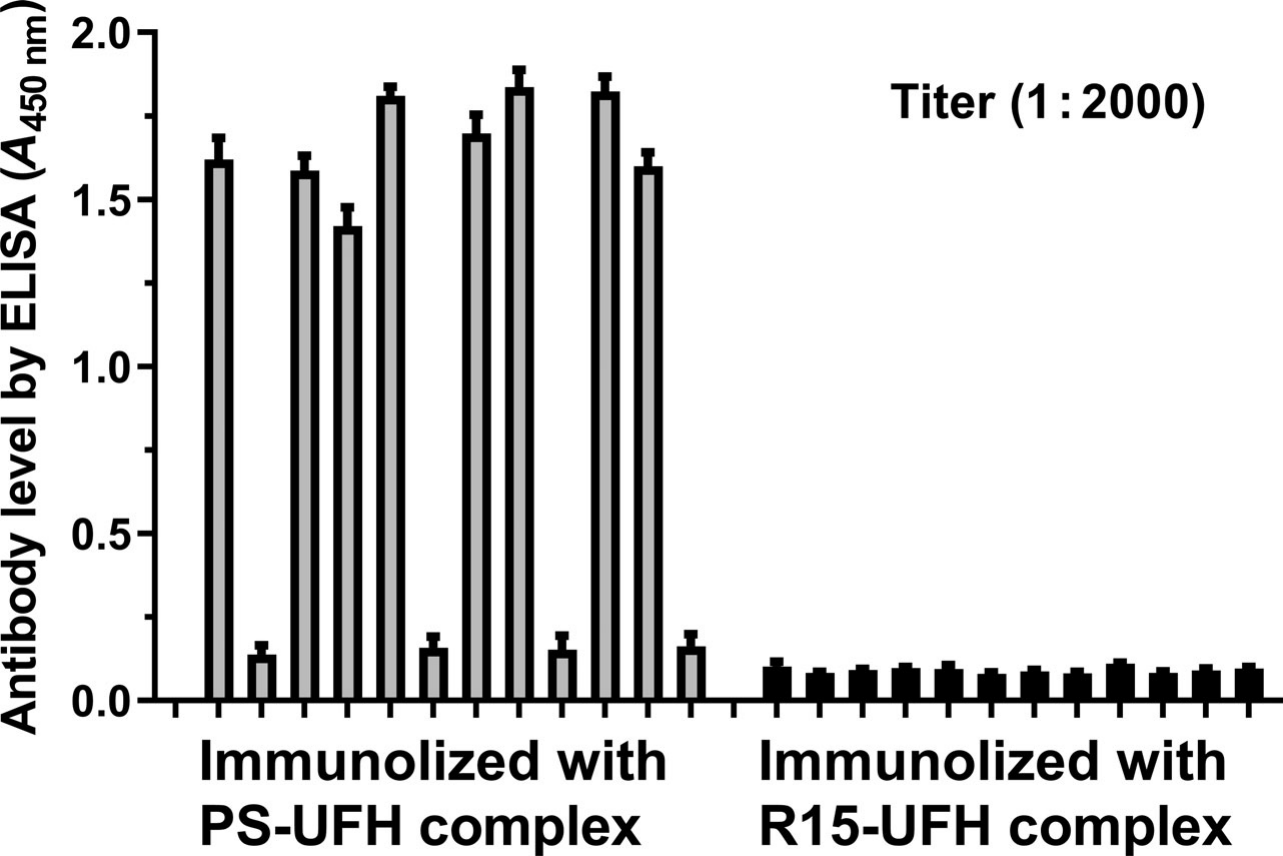
Detection of the UFH–PS and UFH–R15 complexes antibodies with enzyme‐linked immunosorbent assays (ELISA). Guinea pigs (*n* = 12) were immunized using the UFH–PS and UFH–R15 complexes (premixed before immunization). High‐affinity microplates coated with the UFH–PS and UFH–R15 complexes were used to detect antibody levels. Sera were diluted 2000 times with PBS buffer for detection. Data are presented as mean ± SD. The samples were measured with an ELISA method in triplicate.

Guinea pigs were used to evaluate the immunogenicity of the UFH–PS and UFH–R15 complexes. As seen in Fig. 7, all of the guinea pigs survived to the end of the experiment. Overall, 8 of the 12 guinea pigs immunized with UFH–PS complex exhibited high levels of antibodies, whereas none of the 12 guinea pigs immunized with the UFH–R15 complex produced any detectable antibodies. Skin damage on the immune spots or around the nose were observed in the guinea pigs after UFH–PS complex immunization, which was not present in the guinea pigs immunized with UFH–R15 complex.

## Discussion

It has been 153 years since PS was discovered, and PS remains the only approved agent to reverse UFH. The regular needs for UFH reversal include heart transplantation, CABG, repair of congenital heart problems, and cardiac valve repair, and occasional needs include aortic aneurysm repair, PCI, carotid endarterectomy, femoral popliteal bypass, complex vascular reconstruction, and arteriovenous fistula grafts for dialysis [30]. However, the use of PS clinically often includes adverse reactions characterized by pronounced systemic hypotension, refractory ventricular fibrillation, dilated right ventricle and impaired heart contractility, widespread pulmonary infiltrates, acute respiratory distress syndrome, hypoxemia, and mixed acidosis [4, 7, 31]. In addition, the etiology of PS‐induced adverse reactions is multifactorial, and we do not know exactly what causes these side effects. This plight has retarded the design and development of PS substitutes. Given that PS is a series of peptides rich in arginine, the strong alkaline nature promotes the binding of PS to cells or proteins in the blood system, including erythrocytes, fibrinogen, and coagulation factors [32]. In addition, the heterogeneous property and formation of UFH–PS complex are also risk factors associated with the application of PS. Therefore, our studies start from the influences of R15 on erythrocytes, fibrin polymerization, complement activation, and coagulation functions. Animal experiments were performed to explore the potential toxicity of R15 and UFH–R15 complex on the blood system, immune system, and organs. Furthermore, comparisons were made between PS and R15 during all the experiments.

### Potency determination and verification

Three methods were employed and compared to determine the potency of PS and R15 on UFH reversal. APTT assays and anti‐FXa assays were often used to monitor heparin clinically and determine the potency of PS and PS substitutes in preclinical research [22, 23, 24, 25, 26]. It was observed that the binding of PS and R15 to UFH in PBS increased turbidity at 500 nm, and a method depicting turbidity change was applied for potency determination. Three potency determination methods were compared, and we found turbidity assays were the most sensitive. The calculated potency of PS when using the turbidity method was the same as that from anti‐FXa assays, and the results were different from the results observed with APTT assays (Fig. 1A,B). Considering the limitation of APTT in monitoring UFH [33], and the recommendation of anti‐FXa assays in the application of UFH determination [34, 35], the anti‐FXa assays and turbidity assays were chosen to determine the potency of PS and R15, followed by verification in rats *in vivo*.

### Interaction between erythrocytes, fibrin, complement, and rat plasma

The interaction between erythrocytes and polycations depended not only on charges but also on their chemical nature. Polycations, in PS, for instance, neutralize the negative charges on the membrane surface of erythrocytes, leading to hemagglutination [27]. Besides electrostatic interactions, the residues of hydrophobic groups on polycationic peptides and membranes of erythrocytes induce severe changes in the lipid bilayer of erythrocyte membranes, leading to hemolysis or lipid bilayer permeabilization without irreversible disruption of the membrane [36]. Therefore, considering the highly cationic nature, influences of PS and R15 on erythrocytes were investigated using the hemolysis assay, microscope observation of erythrocyte morphology, an erythrocyte aggregation assay, and an erythrocyte osmotic resistance assay. Both PS and R15 can neutralize the surface charges of erythrocyte. However, they do not affect the membrane of erythrocytes at 4 U·mL^−1^ (the mean concentration of UFH expected in a patient undergoing cardiopulmonary bypass surgery) [37].

Fibrinogen, an anionic protein circulating in the blood system, interacts with cationic PS, resulting in disturbance of fibrin polymerization and fibrinolysis [38]. The binding of PS and R15 to fibrinogen increased the initial turbidity (before thrombin addition) and final turbidity of fibrin (end of polymerization), accelerating the process of polymerization and disarraying and thickening fibrin strands concentration‐dependently. R15 in whole blood did not affect fibrin fibers whether in morphology or diameter, whereas the changes in fibrin treated with PS remained.

PS and UFH–PS complex induce activation of complement via the classical complement pathway, generating potent anaphylatoxins C3a and C5a [39]. Although the specific mechanism of PS‐induced pulmonary hypertension remains unknown, there is a strong correlation between complement activation and acute pulmonary syndromes, including catastrophic pulmonary hypertension, increased airway pressure, and pulmonary edema [14, 40]. We found that UFH and R15 lost their complement activation ability when UFH–R15 complex was formed. A confusing phenomenon was observed in that complement was activated by the UFH–PS complex at 0.2 and 0.4 U·mL^−1^ (Table 2). Further studies are warranted to verify and decipher this finding. We also found that when the UFH was neutralized by insufficient PS or R15, the mixture strongly activated the complement. To reveal the possible cause, we calculated the free and total concentrations of the substances in the mixture. In Fig. 5B,C, when UFH is excess, the ability of the UFH–PS complex to activate the complement was 7–8 times stronger than that of the UFH–R15 complex by comparison of CH50. It was still unclear how the UFH‐antagonist complex with excess UFH activated complement even at very low concentrations, at which the UFH alone did not activate complement. It is also unclear whether the difference in the ability of the UFH–PS and UFH–R15 complexes in excess of UFH to activate complement is due to the immunogenicity of PS or other unknown factors. Further studies are needed to decipher this phenomenon. Finally, we investigated activation of complement by the UFH–PS complex and the UFH–R15 complex when PS and R15 were in excess. The results demonstrated that the activation of complement by the UFH–R15 complex with excess R15 may be due to free R15 in the mixture, because R15 alone can activate complement at the same concentrations (Fig. 5E).

PS has anticoagulant properties that have been attributed to the inhibition of coagulation factors involving factor V, factor VII, and factor X [41, 42]. Therefore, we tested the APTT of Wistar rats in the presence of PS and R15. R15, similar to PS, elevated the APTT value of plasma concentration‐dependently. When we extended the incubation time, APTT of plasma with R15 returned to a normal level within 2 h, whereas APTT of plasma treated with PS remained. When we incubated UFH–R15 complex with plasma for 4 h, increases in amount of free UFH were detected by both APTT assays and anti‐FXa assays, suggesting the release of UFH from UFH–R15 complex, indicating the possible enzymatic hydrolysis of R15. This may partially explain the difference in the effect of PS and R15 on fibrin fibers in whole blood. PS‐treated whole blood at 40 U·mL^−1^ did not coagulate, whereas whole blood with R15 regained its function of coagulation within 1 h. In addition, R15 at concentrations of 16 and 40 U·mL^−1^ strongly influenced the fibrin fibers formed by pure fibrinogen. This influence became less obvious in whole blood incubated with R15, which may be due to the degradable property of R15.

The reappearance of UFH after UFH reversal, called heparin rebound, may cause continuous bleeding and excess blood loss [43]. Animal studies show that PS (half‐life for rats: 24 min) is metabolized and excreted by the kidneys, and UFH–PS complex (half‐life for rats: 18 min) is mainly metabolized in the liver [44, 45]. The specific mechanism underlying heparin rebound is still unknown. Considering the potential heparin rebound of R15, two doses of UFH (300 and 1000 U·kg^−1^) were applied to induce heparinization in rats, followed by a corresponding reversal dose of R15 or PS. No signs of heparin rebound were found in two animal experiments, possibly because the clearance of UFH–R15 complex was quicker than the disassociation of R15 from UFH–R15 complex. R15 can be degraded by carboxypeptidase that cleaves off the C‐terminal arginine residue [46]. The main metabolites of R15 were R12, R13, and R14, which also possess the binding ability of UFH [21]. Further studies are needed to monitor heparin rebound in large animals (dogs or monkeys) with extended observation times. In consideration of the blunt degradation of PS in plasma and strong complement activator of UFH–PS complex in the presence of free UFH, the deposit of UFH–PS complex in pulmonary and renal capillaries may be related to PS‐induced pulmonary syndromes, which were thought to be caused by complement activation [47, 48, 49]. Further studies are needed to support this idea.

### Safety studies *in vivo*

To investigate the safety of R15 *in vivo*, three animal experiments were carried out: (a) short‐term toxicity of a single injection of PS or R15 (900 U·kg^−1^) into Wistar rats in the absence or presence of UFH; (b) repeated UFH reversal toxicity in BALB/c mice by PS or R15 (300 U·kg^−1^); and (c) immunogenicity of the UFH–PS and UFH–R15 complexes using guinea pigs. Increased WBCs were found 1 h after a single injection of UFH both in rats and in mice. Therefore, it is uncertain whether UFH–R15 complex or UFH itself elevated WBC. Slight decreases in RBC were observed in heparinized mice at 1 h after UFH reversal by PS and R15 (300 U·kg^−1^), which was not found in heparinized rats reversed by PS and R15 (900 U·kg^−1^). This decrease in RBC was only observed once in three events of short‐term hematological analysis. A total of 900 U·kg^−1^ of PS and R15 injection into rats increased CREA levels, indicating possible kidney damage. This may be due to the cationic PS that could neutralize anionic sites in the glomerulus, causing reversible epithelial damage [50, 51]. In immunogenicity detection experiments, antibodies were detected in guinea pigs immunized with UFH–PS complex, whereas no detectable evidence was found in guinea pigs with UFH–R15 complex immunization.

In conclusion, *in vitro* and *in vivo* safety studies on R15 were undertaken. All experiments demonstrated that UFH neutralization by R15 was safe at therapeutic dose or concentration. The influences of R15, similar to PS, on erythrocytes, fibrin, complement, and the function of rat plasma were concentration‐depended, and these influences weakened or were avoided in whole blood or live animals, which may partially be ascribed to the metabolism of PS and R15. UFH–PS complex possessed immunogenicity that was completely avoided by UFH–R15 complex. We found the dramatically enhanced ability of the UFH–PS complex with excess UFH to activate complement, which may be associated with PS‐induced pulmonary syndromes. This study provides methodological support for the efficacy and safety evaluation of PS substitutes and paves the way for further development and clinical application of arginine‐based heparin antagonists. Our future studies will focus on the mechanism of PS‐induced pulmonary syndromes, the influences of R15 on platelets, and the monitoring of heparin rebound of R15 in dogs or monkeys.

## Materials and methods

### Reagents

PS, complete Freund's adjuvant, and incomplete Freund's adjuvant were purchased from Sigma‐Aldrich (St. Louis, Missouri, US); UFH was purchased from Guoyao Company (Shanghai, China); R15 (supplied as chloride salts) was synthesized by SciLight Biotechnology, LLC (Beijing, China); thrombin, 6% sheep red blood cell, anti‐SRBC hemolysin, and freeze‐dried guinea pig serum were purchased from Solarbio (Beijing, China); fibrinogen was purchased from China Biological Product, LLC (Shandong, China); heparin anti‐FXa kit (reagents inside: Factor Xa, Factor Xa substrate, AT, and buffer) were purchased from Biophen BioMed Company (Neuville‐sur‐Oise, FR); goat anti‐guinea pig IgG‐HRP was purchased from Boaosen, LLC (Beijing, China); goat anti‐mouse IgG‐HPR was purchased from Gene‐star (Beijing, China); total complement determination kit was purchased from Kexing Shangmao, LLC (Shanghai, China); and actin cephaloplastin and calcium chloride were purchased from Siemens Company (Marburg, Germany).

### Animals

Male Wistar rats (250–300 g), female guinea pigs (300–320 g), and male BALB/c mice (8–10 weeks) were purchased from Charles River (Beijing, China). All animals were housed under standard conditions (temperature: 25 ± 3 °C, humidity: 50 ± 5%, and 12‐h light–dark cycle) for at least 7 days before the experiments. All animal procedures were approved and reviewed by the National Institutes of Health Guide for the Care and Use of Laboratory Animals and the regulations derived by the Animal Care and Welfare Committee of the Institute of Radiation Medicine, Academy of Military Medical Sciences (Beijing, China; IACUC‐DWZX‐2020‐503).

### Concentration and dose unit of UFH, PS, and R15 in the study

PS and PS‐like chemicals with different positive charges are not only related to the UFH reversal ability, but are also related to PS‐induced toxicity [52]. The determined potencies of PS and R15 were 150 and 170 U·mg^−1^, suggesting that each mg of PS or R15 can neutralize 150 or 170 U of UFH, respectively. The unit of U was mainly used as the concentration and dose unit of PS and R15 in our experiments to facilitate the comparison between PS and R15 at the same efficacy. All dilutions and concentrations are final unless otherwise specified.

For example, PS (X U·mL^−1^) represents the amounts of PS per mL that can neutralize exactly X U of UFH. When PS was added to a solution or matrix comprising UFH, it is expressed as UFH–PS (Y U·mL^−1^; Z U·mL^−1^), indicating that the final concentrations of PS and UFH in the solution or matrix is Y and Z U·mL^−1^, respectively. When Y is equal to Z, UFH is exactly neutralized by PS. When Y is higher than Z, PS is thought to neutralize UFH to form UFH–PS complex with excess PS and *vice versa*.

### Therapeutic concentration and dose of UFH in the study

Although there is no clear and accurate method for the calculation of the initial UFH dose for patients in need of cardiopulmonary bypass (CPB), 300 U·kg^−1^ of UFH is recommended as the optional dose for maintaining activated clotting time (ACT) of more than 400 s [53]. 4 U·mL^−1^ was considered as the mean concentration of UFH expected for a patient undergoing CPB [28]. Accordingly, 4 U·mL^−1^ of UFH was used as the therapeutic concentration in our *in vitro* studies. In our animal experiments, 300 U·kg^−1^ of UFH was used as the therapeutic dose; that is, to neutralize 300 U·kg^−1^ of UFH, we assumed that 300 U·kg^−1^ of PS or R15 was needed.

### Potency determination *in vitro*

Three assay methods, namely, APTT, anti‐FXa, and turbidity assays, were applied to determine the potency of PS and R15 for UFH reversal. In those methods, fixed concentrations of UFH were neutralized using increasing concentrations of PS and R15 each. In APTT assays, a modified procedure was followed [54] wherein 15 μL of UFH (4 U·mL^−1^, final) in Wistar rat plasma was reversed using 15 μL of increasing concentrations of PS and R15 each and the APTT clotting time was measured after adding actin cephaloplastin and calcium chloride. In anti‐FXa assays, UFH (1 U·mL^−1^, final) was reversed by PS and R15 each in increasing concentrations aided by a heparin anti‐FXa kit. In turbidity assays, UFH (4 U·mL^−1^, final) in PBS was reversed by adding increasing concentrations of PS and R15 each. The samples were vortexed and allowed to stand at room temperature for 5 min. Then, the absorbance of the mixtures at 500 nm was measured using a microplate reader (Spectra Max 190, Molecular Devices, Silicon Valley, California, USA).

### Potency verification in rats *in vivo*

A modified procedure that was described elsewhere was followed [21]. In brief, 24 Wistar rats were randomized into 4 groups (six rats per group). Rats were anesthetized with a mixture of isoflurane and oxygen. A total of 300 U·kg^−1^ of PS or R15 was injected through the tail vein of rats 3 min after the intravenous injection of UFH (300 U·kg^−1^). A total of 0.5 mL of blood was collected from the heart into a tube containing 3.8% of sodium citrate at 5 min after the injection of PS or R15. The blood samples were centrifuged at 5867 g·min^−1^ for 10 min, and the APTT assays were performed immediately. Rats injected with UFH and saline alone were taken as the positive and negative control separately.

### Hemolysis and morphology of erythrocytes

Two microliters of citrated whole blood that was drawn from the heart of Wistar rats was incubated at 37 °C for 1 h with 198 μL of either PS or R15 with different concentrations (100 to 5000 μg·mL^−1^). The mixtures were centrifuged for 10 min at 1000 g·min^−1^, and the supernatants were analyzed at 540 nm with a microplate reader. PBS and 1% Triton were used as negative and positive controls, respectively. The percentages of erythrocyte hemolysis were obtained from this Eqn (1):

(1) $$Y\;\left(\%\right)=(\left(A_{\text{sample}}‐A_{\text{negative}}\right)/\left(A_{\text{positive}}‐A_{\text{negative}}\right))\times 100\%$$

The same procedures abovementioned were followed to obtain the erythrocytes incubated with different concentrations of PS or R15 (50–5000 μg·mL^−1^). A total of 10 μL of the mixtures was applied to erythrocyte morphology study using an optical microscope (XDS‐1B, COIC, Chongqing, China).

### Erythrocyte osmotic resistance assay

A total of 108 μL of whole blood (4% EDTA‐2K as anticoagulant) drawn from the heart of Wistar rats was incubated with 12 μL of varying concentrations of PS or R15 at 37 °C for 1 h. Then, 10 μL of the mixtures was added to the saline solutions at nine different concentrations, ranging from 0.2% to 0.8% g·mL^−1^, at room temperature. After incubation for 30 min, the mixtures were centrifuged at 1000 g·min^−1^ for 5 min. Finally, the hemoglobin levels were measured at 540 nm using a microplate reader. NaCl solutions at 0.9% g·mL^−1^ and 0% g·mL^−1^ were set as 0% and 100% hemolysis, respectively. Erythrocyte hemolysis percentages were calculated using Eqn (1), and the results were presented as the mean osmotic resistance (MOR50), which is the concentration of NaCl at which 50% of the erythrocytes were lysed.

### Erythrocyte aggregation assay

Blood collected from the heart of Wistar rats was anticoagulated by 4% EDTA‐2K. Some of the blood was centrifuged at 5867 g·min^−1^ for 10 min, and platelet‐poor plasma (PPP) was achieved to adjust hematocrit (HCT) to 40%. Then, 100 μL of the test substances (PS, R15, and UFH–PS and UFH–R15 complexes) with varying concentrations was incubated with 1000 μL of HCT‐adjusted blood for 1 h at 37 °C. The degree of erythrocyte aggregation was measured with a rheometer (BT‐300; Bolaitetong, Beijing, China). The results were presented as the aggregation index (AI) calculated with Eq (2) [55]:

(2) $$\text{AI}=\eta_{L}/\eta_{H}$$

where *η* represents apparent viscosity, *η*_L_ is the apparent viscosity at a low shear rate (1 s^−1^), and *η*_H_ is the apparent viscosity at a high shear rate (200 s^−1^).

### Fibrin polymerization assay

HEPE buffer solution (20 mm HEPEs with 150 mm NaCl) was prepared and used to dilute fibrinogen, PS, UFH, and R15. Then, 150 μL of fibrinogen (1 mg·mL^−1^), 20 μL of CaCl_2_ (25 mm), and different concentrations of test substances (PS, R15, and UFH–PS and UFH–R15 complexes) were mixed at 37 °C for 10 min. Fibrin polymerization was initiated by adding 20 μL of thrombin (25 U·mL^−1^). The polymerization curves were constructed by recording the changes in the optical density at 405 nm every 30 s for 1 h at 37 °C using a microplate reader. The time taken for half maximal turbidity (TMT50) was calculated on the basis of these fibrin polymerization curves. The assay was repeated thrice, and the average TMT50 value was reported.

### Scanning electron microscopy of pure fibrin and whole blood clots

Fibrin was prepared for scanning electron microscopy (SEM) as per the procedure mentioned above. Briefly, fibrinogen was incubated with varying concentrations of PS or R15 and CaCl_2_ at 37 °C for 10 min. The transformation from fibrinogen to fibrin was initiated by adding thrombin. After incubation for 1 h at 37 °C, the clots were washed thrice with HEPEs buffer and fixed overnight using 2.5% glutaraldehyde, after which the clots were washed with deionized water thrice. The clots were then dehydrated using ethanol/water (50/50 to 100/0, v/v) and dried using a CO_2_ critical point dryer. Images were captured at magnifications of 5000× and 10 000× from different areas of gold sputter‐coated clots. The diameters of the fibrin fibers were measured using imagej software (National Institute of Health, Bethesda, MD, USA).

The influences of PS, R15, and UFH–PS and UFH–R15 complexes on fibrin in whole blood were also determined. A total of 170 μL of citrated whole blood drawn from the heart of Wistar rats was incubated with 20 μL of CaCl_2_ (25 mm) and 10 μL of test substances (PS, R15, and UFH–PS and UFH–R15 complexes) at 37 °C in silicone‐coated tubes, and blood clots were acquired 20 min later. The same procedures were applied for the preparation of whole blood clots for SEM. The diameters of the fibrin fibers in the clots were measured using imagej software.

### Complement activation assay

The influence of PS and R15 on complement was investigated with a modified hemolytic complement assay using the sera of guinea pigs [56]. Six percent of sheep red blood cells (SRBCs) were washed with TEAE buffer (0.21 m triethanolamine, 0.18 m HCl, 1.28 m NaCl, 4.9 mm MgCl_2_, and 1.4 mm CaCl_2_). Six percent of sensitive SRBCs was achieved by mixing 6% SRBC with anti‐SRBC hemolysin for 30 min at 37 °C. Then, 200 μL of PS or R15 with increasing concentrations was incubated with 400 μL of diluted guinea pig sera at 37 °C for 30 min. The mixtures were then centrifuged at 367 g·min^−1^ for 10 min, and the supernatant was pipetted into a 96‐well plate to read the absorbance of 541 nm on a microplate reader. TEAE buffer with or without guinea pig serum was set as negative (100%) and positive controls (0%), respectively. The level of complement remaining in sera samples was calculated with Eqn (3) as follows:

(3) $$Y\;\left(\%\right)=\left(A_{\text{sample}}‐A_{0\%}\right)/\left(A_{100\%}‐A_{0\%}\right)\times 100\%$$

The complement level–concentration curves were depicted, and CH50 (concentration at which 50% of the SRBCs were lysed) was achieved.

We also evaluated the ability of the UFH–PS and UFH–R15 complexes to activate complement in the sera of guinea pigs. A total of 150 μL of UFH (0.24–96 U·mL^−1^, prepared concentration) was neutralized with 150 μL of PS or R15 (0.24–96 U·mL^−1^, prepared concentration). A total of 200 μL of the mixture was added to 400 μL of diluted sera at 37 °C for 30 min. The final concentration of the UFH–PS and UFH–R15 complexes in the sera samples was 0.04–16 U·mL^−1^, and the complement levels were determined by the procedures mentioned above. Then, we adjusted the mixing ratio of UFH antagonists (PS and R15) to UFH to allow excess antagonists or UFH in the UFH‐antagonist complex solutions. To acquire excess UFH, 160 μL of UFH (0.24–96 U·mL^−1^, prepared concentration) was mixed with 140 μL of PS or R15 (0.24–96 U·mL^−1^, prepared concentration). To acquire excess antagonists, 140 μL of UFH (0.24–96 U·mL^−1^, prepared concentration) was mixed with 160 μL of PS or R15 (0.24–96 U·mL^−1^, prepared concentration).

UFH was considered theoretically unbound when it was not fully neutralized by PS or R15. PS or R15 was considered theoretically unbound when UFH was neutralized by excess PS or R15. Total UFH was the total amount of UFH in the solution, regardless of the presence of a binding antagonist. The final concentration of free UFH, free PS, or free R15 in sera was calculated with Eq (4):

(4) $$C_{\left(\text{free}\;\text{conc}.\right)}=C_{\left(\text{prepared}\;\text{conc}.\right)}/45$$

where *C*_(prepared conc.)_ is the prepared concentration of UFH, PS, or R15 before mixing. *C*_(free conc.)_ was the theoretical concentration of unbound UFH, PS, or R15 in the sera.

The final concentration of total UFH, total PS, or total R15 in sera was calculated with Eqn (5):

(5) $$C_{\left(\text{total}\;\text{conc}.\right)}=C_{\left(\text{prepared}\;\text{conc}.\right)}\times 8/45$$

where *C*_(prepared conc.)_ is the prepared concentration of UFH, PS, or R15 before mixing. *C*_(total conc.)_ is the final concentration of UFH, PS, or R15 in sera, whether bounded or not.

### Interaction with rat’s plasma *in vitro*

We investigated the influence of PS and R15 on coagulation functions of Wistar rat plasma. Three factors were considered: (a) concentrations of test substances; (b) exposure time to plasma; and (c) the absence or presence of UFH. First, we investigated the influence of PS and R15 with varying concentrations on the plasma of Wistar rats using APTT assays. PS and R15 at different concentrations were added into the plasma, followed by 2‐h incubation at 37 °C. Samples were collected at different time points and measured with APTT assays immediately. Plasma with saline was set as a control. Secondly, the influence of the UFH–PS and UFH–R15 complexes with varying concentrations on plasma was investigated. Varying concentrations of UFH in plasma were exactly neutralized with PS and R15, followed by 4‐h incubation at 37 °C. Samples from different time points were measured with APTT assays and anti‐FXa assays simultaneously.

### UFH monitoring in heparinized rats reversed by PS or R15

Twenty‐four male Wistar rats were divided into four groups: (a) saline group (only saline was given); (b) UFH group (only UFH was given); (c) PS reversal group (UFH was given followed by PS reversal); and (d) R15 reversal group (UFH was given followed by R15 reversal). The rats were anesthetized using 3% pentobarbital and cannulated through the left jugular artery for blood collection. UFH (300 U·kg^−1^) was injected through the tail vein, followed by PS or R15 reversal (300 U·kg^−1^). Blood samples were collected from the left jugular artery every 30 min up to 4 h. Blood samples were anticoagulated by 3.8% sodium citrate at the ratio of 9 : 1, followed by centrifugation of 5867 g·min^−1^ for 10 min at 4 °C. The plasma samples were acquired and stored at −80 °C for APTT and anti‐FXa assays. Next, we increased the dose of UFH from 300 to 1000 U·kg^−1^, and the same procedures were followed except that only anti‐FXa assays were performed for sample measurements.

### Short‐term toxicity in rats

Twenty‐four male Wistar rats were randomly divided into six groups (four rats per group) and anesthetized with a mixture of isoflurane/oxygen mixture: (a) saline group (only saline was given); (b) UFH group (only UFH was given); (c) PS group (only PS was given); (d) R15 group (only R15 was given); (e) PS reversal group (UFH was given followed by PS reversal); and (f) R15 reversal group (UFH was given followed by R15 reversal). The dose of UFH, PS, or R15 injected into the rats of each group was all 900 U·kg^−1^. The saline group was used as the control. For each group, blood samples of rats were collected from the heart at 1 h after administration of the test substances, followed by hematological and biochemistry analysis, determination of complement level, and APTT assays. The levels of total complement in the sera samples of rats were determined with a total complement determination kit. Fragments of organs (heart, liver, spleen, lung, and kidney) were harvested and fixed in 10% formalin, embedded in paraffin, and sectioned for hematoxylin and eosin (H&E) staining. The lung alveolar area from the images of lung sections was measured with imagej software. The analyzed hematological parameters included the following: white blood cells (WBCs), hemoglobin (HGB), hematocrit (HCT), mean corpuscular hemoglobin (MCH), mean corpuscular volume (MCV), mean corpuscular hemoglobin concentration (MCHC), red blood cells (RBCs), and platelets (PLTs). Biochemical parameters included the following: aspartate aminotransferase (AST), creatinine (CREA), alkaline phosphatase (ALP), alanine aminotransferase (ALT), and creatine kinase (CPK).

### Repeated UFH reversal in BALB/c mice

Sixty‐four female BALB/c mice aged 8–10 weeks were randomly divided into four groups (16 rats per group): (a) saline group (only saline was given); (b) UFH group (only UFH was given); (c) PS reversal group (PS was given 3 min after UFH injection); and (d) R15 reversal group (R15 was given 3 min after UFH injection). The doses for UFH, PS, and R15 were all 300 U·kg^−1^. The heparinization/neutralization regimen was that all test substances were given through the tail vein once a week for five weeks (day 1 and days 8, 15, 22, and 29). During the whole experiment, the body weight of the mice in each group (16/16) was recorded once a week for five weeks. Six of 16 mice in each group were under hematological analysis at 1 h after administration of the test substances at the 1^st^, 3^rd^, and 5^th^ week (day 1 and days 15 and 29). At the 6^th^ week (day 36), the rest of the mice in each group (never bled for hematological analysis previously) were subjected to hematological analysis. Then, all mice were sacrificed and the organs (heart, liver, spleen, lung, and kidney) were harvested for histology examination. The lung alveolar area from the images of lung sections was measured with imagej software. Blood samples from each mouse were collected for antigenicity evaluation.

An enzyme‐linked immunosorbent assay (ELISA) was used for immunogenicity evaluation [57]. Briefly, the UFH–PS (1 U·mL^−1^; 1 U·mL^−1^) and UFH–R15 (1 U·mL^−1^; 1 U·mL^−1^) complexes were coated onto a high‐affinity 96‐well plate and incubated at 4 °C overnight. The wells were blocked using 1% BSA and incubated at 37 °C for 1.5 h. Diluted sera (1 : 50) from each mouse were added to the wells and then incubated at 37 °C for 2 h. Then, goat anti‐mouse IgG‐HRP and TMB substrates were used to detect the level of antibodies produced in the mouse sera.

### Immunogenicity of the UFH–PS and UFH–R15 complexes

Guinea pigs were used to evaluate the immunogenicity of the UFH–PS and UFH–R15 complexes. Female guinea pigs were immunized with UFH–PS complex or UFH–R15 complex in complete Freund’s adjuvant (*n* = 12 for each group). The first booster and second booster in incomplete Freund’s adjuvant were given at the 28^th^ and 42^nd^ day. After 2 weeks, blood samples of the guinea pigs were collected.

For immunogenicity evaluation, the UFH–PS (1 U·mL^−1^; 1 U·mL^−1^) and UFH–R15 (1 U·mL^−1^; 1 U·mL^−1^) complexes were coated onto a high‐affinity 96‐well plate and incubated at 4 °C overnight. The wells were blocked using 2% BSA and incubated at 37 °C for 1.5 h. Diluted sera (1 : 2000) from each guinea pig were added, and the plate was incubated at 37 °C for 30 min. Goat anti‐guinea pig IgG‐HRP and TMB substrates were used to detect the level of antibodies produced in the sera of guinea pigs.

### Statistical analysis

In the study, *n* refers to the number of animals in each experimental group. All tests were performed at least in triplicate. Data are presented as the median with range, and data were analyzed with graphpad prism 8 (GraphPad Software Inc., San Diego, CA, USA) using a nonparametric Kruskal–Wallis test. Analysis of variance was applied to analyze the data and presented as the mean ± SD whenever the data passed a normality test. MOR50, TMT50, and CH50 were calculated from curves fitted by nonlinear regression with four parameters using graphpad prism 8. A *P* value < 0.05 was considered statistically significant.

## Conflicts of interest

The authors declare no conflict of interest.

## Author contributions

TL was in charge of each experiment and the paper writing. ZYM participated in the subject design. XXZ performed animal sample analysis and data analysis. HG participated in the erythrocyte aggregation experiment. RLG participated in the fibrin polymerization experiment. ZNW participated in the erythrocyte hemolysis experiment. TYL managed animal studies, and performed animal sample analysis and data analysis. PH managed animal studies. JRG performed animal sample analysis. SH was a technological assessor of the whole subject. GFD was the organizer and designer of this subject.

## Supporting information

**Fig. S1**. Optical microphotographs of erythrocytes incubated with different concentrations of PS or R15 for 1 h at 37°C.
**Fig. S2**. Characteristics of pure fibrin formed by adding thrombin and CaCl_2_ into fibrinogen in the presence of PS and R15 with increasing concentrations.
**Fig. S3**. Characteristics of whole blood fibrin of Wistar rats formed in blood in the presence of PS and R15.
**Fig. S4**. Influence of PS and R15 on total complement activity and coagulation function of Wistar rats.
**Fig. S5**. Microscopic observation of heart from Wistar rats at 1 h after drug administration.
**Fig. S6**. Microscopic observation of liver from Wistar rats at 1 h after drug administration.
**Fig. S7**. Microscopic observation of spleen from Wistar rats at 1 h after drug administration.
**Fig. S8**. Microscopic observation of lung from Wistar rats at 1 h after drug administration.
**Fig. S9**. Microscopic observation of kidney from Wistar rats at 1 h after drug administration.
**Fig. S10**. Percentage of lung alveolar areas of Wistar rats.
**Fig. S11**. Mean body weight of Balb/c mice (n = 16 mice per group) from baseline to day 29.
**Fig. S12**. Microscopic observation of heart from Balb/c mice at 6^th^ week.
**Fig. S13**. Microscopic observation of liver from Balb/c mice at 6^th^ week.
**Fig. S14**. Microscopic observation of spleen from Balb/c mice at 6^th^ week.
**Fig. S15**. Microscopic observation of lung from Balb/c mice at 6^th^ week.
**Fig. S16**. Microscopic observation of kidney from Balb/c mice at 6^th^ week.
**Fig. S17**. Percentage of lung alveolar areas of Balb/c mice.
**Fig. S18**. Detection of UFH‐PS complex and UFH‐R15 complex antibodies by ELISA assays.
**Table S1**. Vital organ index of Wistar rats treated with test substances (n = 4 rats per group).

## Data Availability

The data that support the findings of this study are available in figures, tables, and the supplementary material of this article.
