# Supplementary material for: In vitro and in vivo safety studies indicate that R15, a synthetic polyarginine peptide, could safely reverse the effects of unfractionated heparin
Source: FEBS Open Bio. 2021 Aug 12;11(9):2468–89. doi: 10.1002/2211-5463.13240 (PMC8409304; doi:10.1002/2211-5463.13240)
Supplement: Supplementary file 1 — Fig. S1. Optical microphotographs of erythrocytes incubated with different concentrations of PS or R15 for 1 h at 37°C. Fig. S2. Characteristics of pure fibrin formed by adding thrombin and CaCl2 into fibrinogen in the presence of PS and R15 with increasing concentrations. Fig. S3. Characteristics of whole blood fibrin of Wistar rats formed in blood in the presence of PS and R15. Fig. S4. Influence of PS and R15 on total complement activity and coagulation function of Wistar rats. Fig. S5. Microscopic observation of heart from Wistar rats at 1 h after drug administration. Fig. S6. Microscopic observation of liver from Wistar rats at 1 h after drug administration. Fig. S7. Microscopic observation of spleen from Wistar rats at 1 h after drug administration. Fig. S8. Microscopic observation of lung from Wistar rats at 1 h after drug administration. Fig. S9. Microscopic observation of kidney from Wistar rats at 1 h after drug administration. Fig. S10. Percentage of lung alveolar areas of Wistar rats. Fig. S11. Mean body weight of Balb/c mice (n = 16 mice per group) from baseline to day 29. Fig. S12. Microscopic observation of heart from Balb/c mice at 6th week. Fig. S13. Microscopic observation of liver from Balb/c mice at 6th week. Fig. S14. Microscopic observation of spleen from Balb/c mice at 6th week. Fig. S15. Microscopic observation of lung from Balb/c mice at 6th week. Fig. S16. Microscopic observation of kidney from Balb/c mice at 6th week. Fig. S17. Percentage of lung alveolar areas of Balb/c mice. Fig. S18. Detection of UFH‐PS complex and UFH‐R15 complex antibodies by ELISA assays. Table S1. Vital organ index of Wistar rats treated with test substances (n = 4 rats per group). [file FEB4-11-2468-s001.pdf]

In vitro and *in vivo* safety studies on a synthetic poly-arginine peptide having unfractionated heparin reversal activity

Tong Li, Zhiyun Meng, Xiaoxia Zhu, Hui Gan, Ruolan Gu, Zhuona Wu, Taoyun Liu, Peng Han, Jiarui Gao, Su Han, and Guifang Dou*

Affiliation of the authors: Department of Pharmaceutical Sciences, Beijing Institute of Radiation Medicine, Beijing, 100850, People's Republic of China.

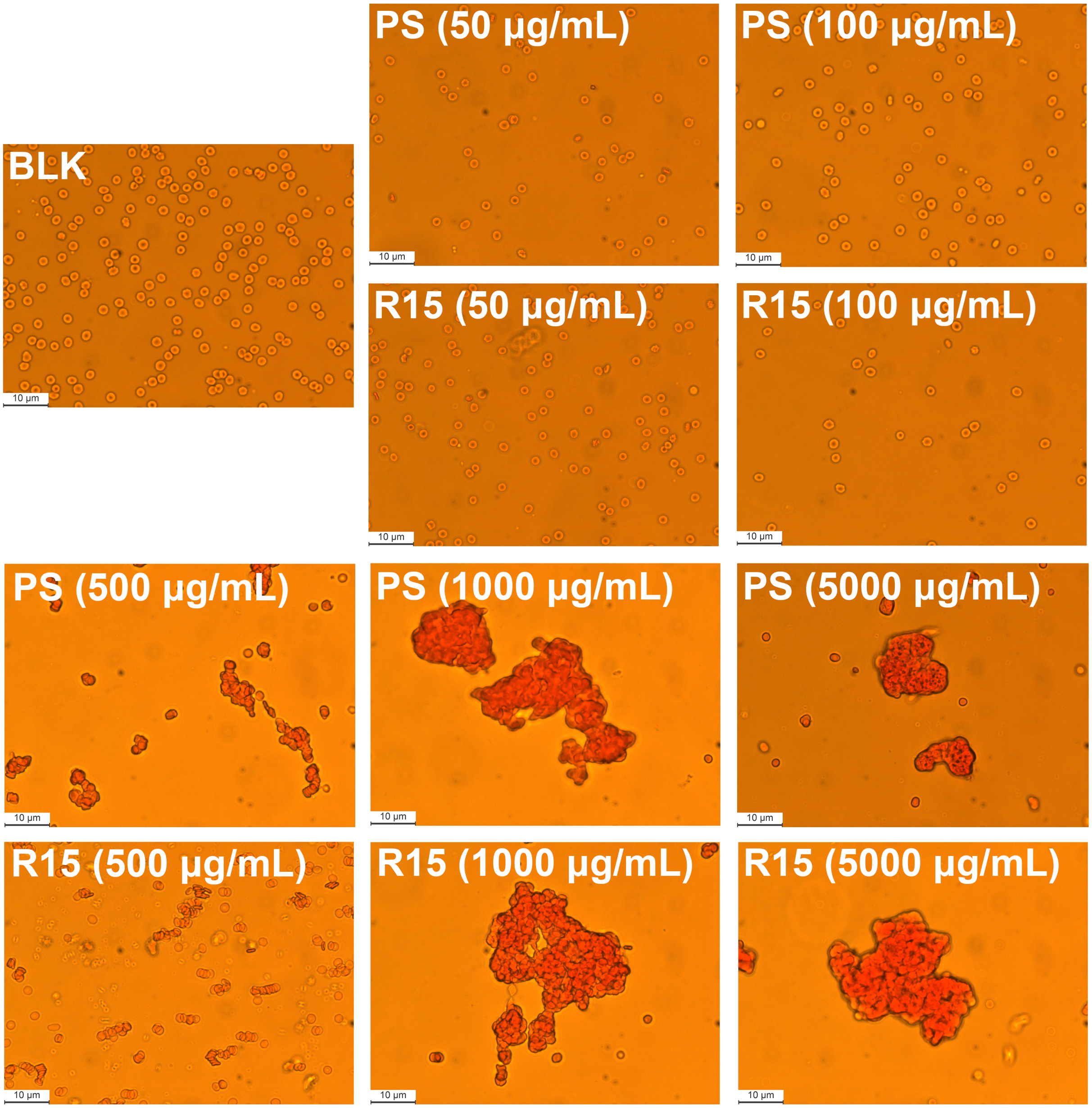

Figure S1. Optical microphotographs of erythrocytes incubated with different concentrations of PS or R15 for 1 h at 37°C. 50, 100, 500, 1,000, and 5,000 µg·mL^{-1} of PS and R15 were tested. BLK represents PBS-treated erythrocytes taken as control. The scale bar is 10 µm.

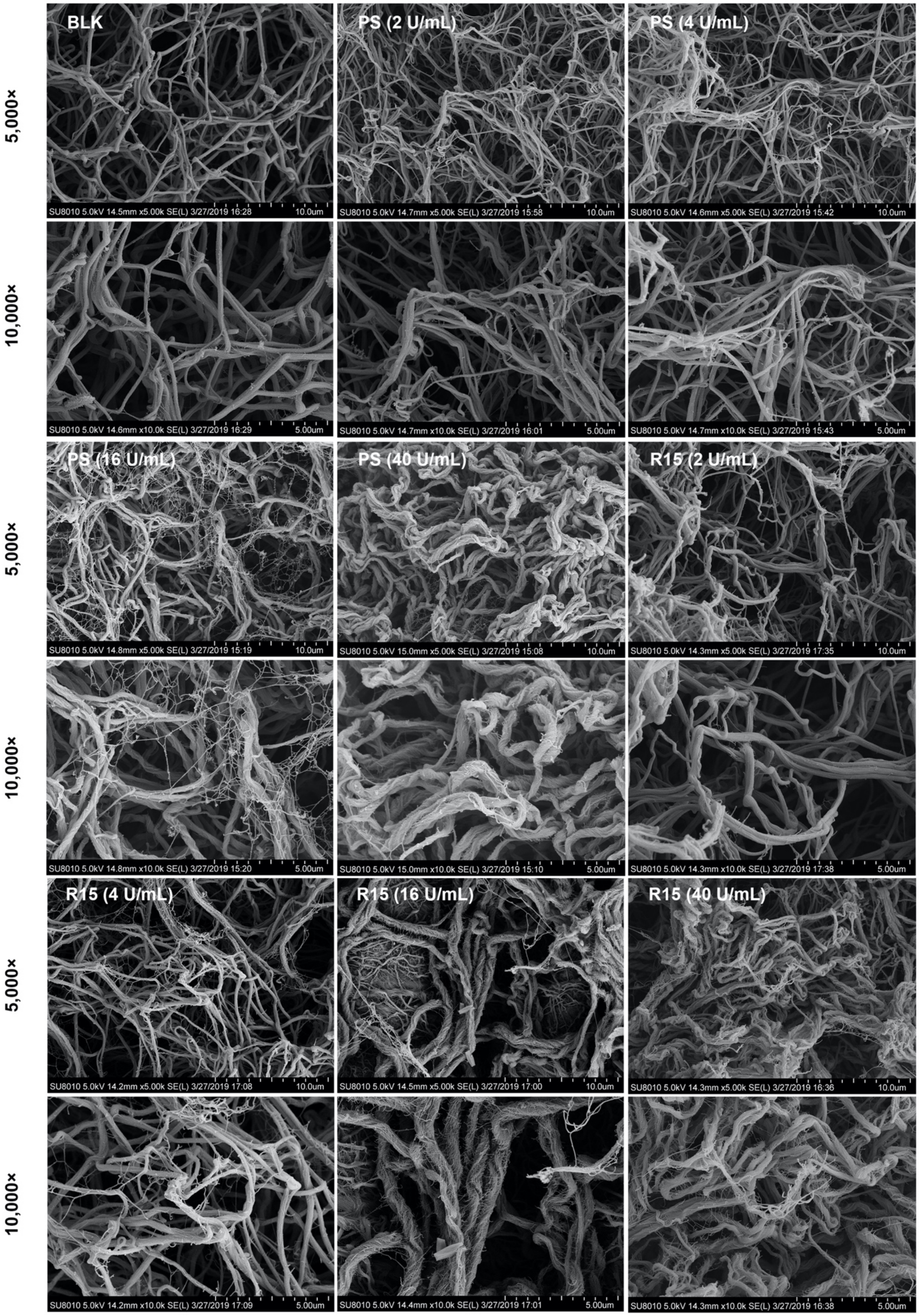
5,000×
10,000×
5,000×
10,000×
5,000×
10,000×
BLK
PS (2 U/mL)
PS (4 U/mL)
PS (16 U/mL)
PS (40 U/mL)
R15 (2 U/mL)
R15 (4 U/mL)
R15 (16 U/mL)
R15 (40 U/mL)

Figure S2. Characteristics of pure fibrin formed by adding thrombin and $CaCl_2$ into fibrinogen in the presence of PS and R15 with increasing concentrations. Images of all clots were captured from different areas at 5,000× and 10,000× magnifications. PS and R15 at concentrations of 2 U·mL^{-1}, 4 U·mL^{-1}, 16 U·mL^{-1}, and 40 U·mL^{-1} were tested. BLK represents HEPEs buffer-treated fibrin taken as control. Both PS and R15 at a concentration of 16 U·mL^{-1} or greater thickened the fibrin fibers and twisted fibrin strands in an irregular manner, which was not found at a concentration of 4 U·mL^{-1} or lower. The scale bar is 10 μm (5,000× magnification) and 5 μm (10,000× magnification), respectively.

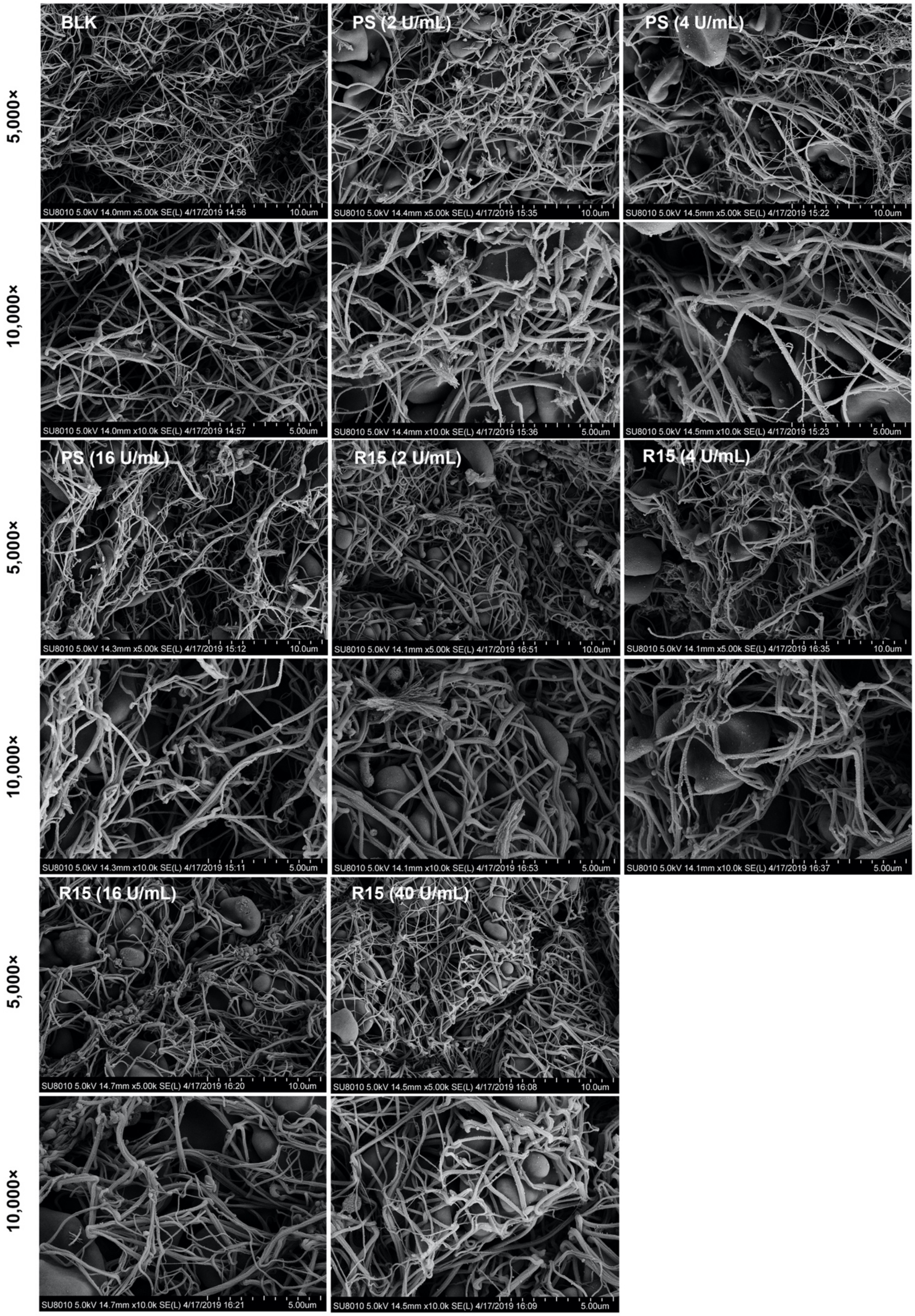
5,000×
10,000×
5,000×
10,000×
5,000×
10,000×
BLK
PS (2 U/mL)
PS (4 U/mL)
PS (16 U/mL)
R15 (2 U/mL)
R15 (4 U/mL)
R15 (16 U/mL)
R15 (40 U/mL)
SU8010 5.0kV 14.0mm x5.00k SE(L) 4/17/2019 14:56 10.0um
SU8010 5.0kV 14.4mm x5.00k SE(L) 4/17/2019 15:35 10.0um
SU8010 5.0kV 14.5mm x5.00k SE(L) 4/17/2019 15:22 10.0um
SU8010 5.0kV 14.0mm x10.0k SE(L) 4/17/2019 14:57 5.00um
SU8010 5.0kV 14.4mm x10.0k SE(L) 4/17/2019 15:36 5.00um
SU8010 5.0kV 14.5mm x10.0k SE(L) 4/17/2019 15:23 5.00um
SU8010 5.0kV 14.3mm x5.00k SE(L) 4/17/2019 15:12 10.0um
SU8010 5.0kV 14.1mm x5.00k SE(L) 4/17/2019 16:51 10.0um
SU8010 5.0kV 14.1mm x5.00k SE(L) 4/17/2019 16:35 10.0um
SU8010 5.0kV 14.3mm x10.0k SE(L) 4/17/2019 15:11 5.00um
SU8010 5.0kV 14.1mm x10.0k SE(L) 4/17/2019 16:53 5.00um
SU8010 5.0kV 14.1mm x10.0k SE(L) 4/17/2019 16:37 5.00um
SU8010 5.0kV 14.7mm x5.00k SE(L) 4/17/2019 16:20 10.0um
SU8010 5.0kV 14.5mm x5.00k SE(L) 4/17/2019 16:08 10.0um
SU8010 5.0kV 14.7mm x10.0k SE(L) 4/17/2019 16:21 5.00um
SU8010 5.0kV 14.5mm x10.0k SE(L) 4/17/2019 16:09 5.00um

Figure S3. Characteristics of whole blood fibrin of Wistar rats formed in blood in the presence of PS and R15. Images of all clots were captured from different areas at 5,000× and 10,000× magnifications. PS and R15 at concentrations of 2 U·mL^{-1}, 4 U·mL^{-1}, 16 U·mL^{-1} and 40 U·mL^{-1} were tested. BLK represents HEPEs buffer-treated whole blood fibrin taken as control. Whole blood treated with PS at the concentration of 40 U·mL^{-1} did not coagulate within 2 h. The scale bar is 10 µm (5,000× magnification) and 5 µm (10,000× magnification), respectively.

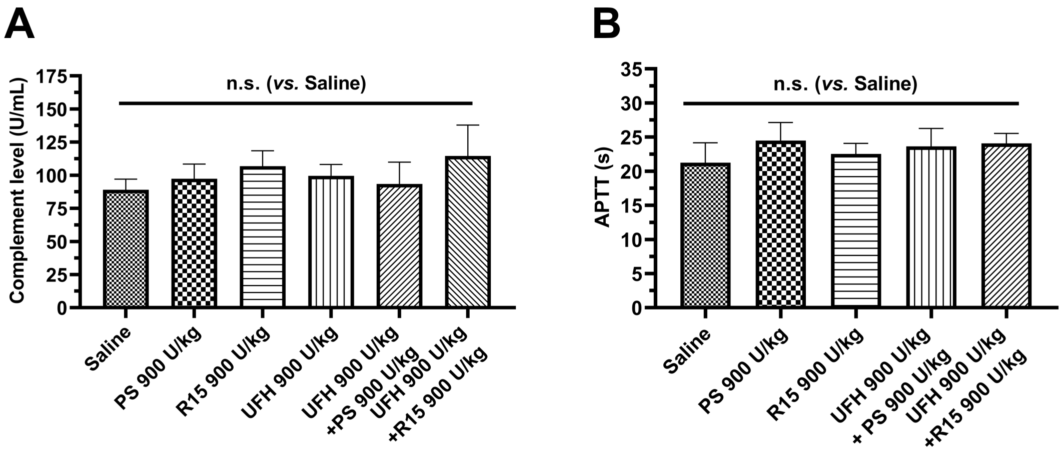

Figure S4. Influence of PS and R15 on total complement activity and coagulation function of Wistar rats. Short-term toxicity was investigated in heparinized or non-heparinized Wistar rats treated with PS or R15 (n=4 rats in each group). Whole blood samples were collected from Wistar rats at 1 h after IV injection of test substances. Rats injected with saline were taken as control. Part of blood samples were anticoagulated (3.8% sodium citrate) and processed for total complement activity determination. Part of blood samples were processed for evaluation of coagulation function using APTT assays. (A) Influences of test substances on total complement activity measured with a total complement determination kit. (B) Coagulation function of Wistar

rats evaluated with APTT assays. The data are expressed as mean ± SD, analyzed by a one-way ANOVA and Dunnett's multiple comparisons test. n.s. represents $p > 0.05$.

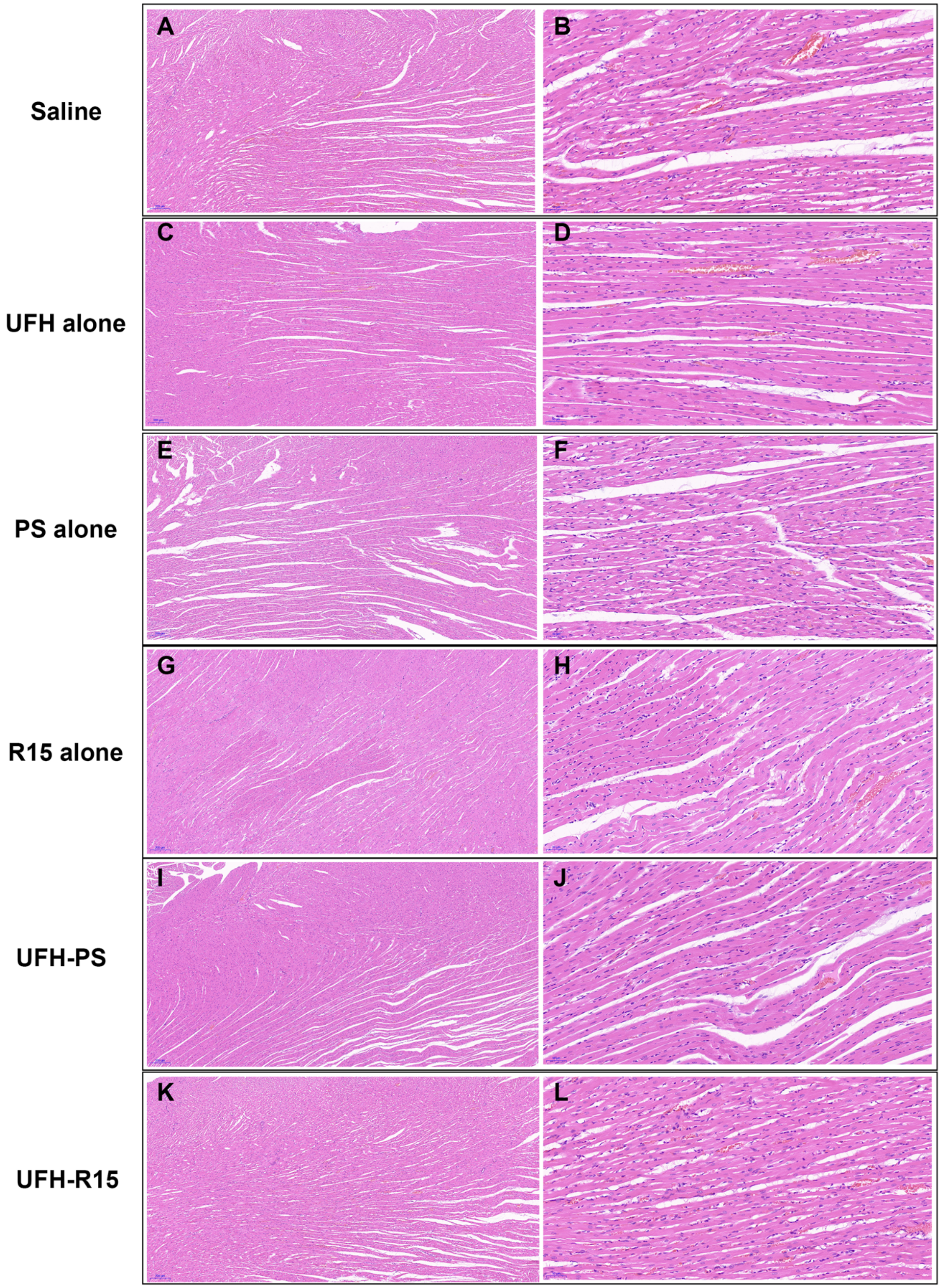
Saline
UFH alone
PS alone
R15 alone
UFH-PS
UFH-R15
A
B
C
D
E
F
G
H
I
J
K
L

Figure S5. Microscopic observation of heart from Wistar rats at 1 h after drug administration. Photomicrographs of histological sections of heart were from Wistar rats at 1 h after treatment with saline (A and B), UFH 900 U·kg^{-1} (C and D), PS 900 U·kg^{-1} (E and F), R15 900 U·kg^{-1} (G and H), UFH 900 U·kg^{-1} + PS 900 U·kg^{-1} (I and J), and UFH 900 U·kg^{-1} + R15 900 U·kg^{-1} (K and L). Saline-treated rats were taken as a control group. Images (A, C, E, G, I and K) were taken using the 5× magnification and images (B, D, F, H, J, and L) were taken using 20× magnification. The scale bar is 200 µm (left column) and 50 µm (right column), respectively.

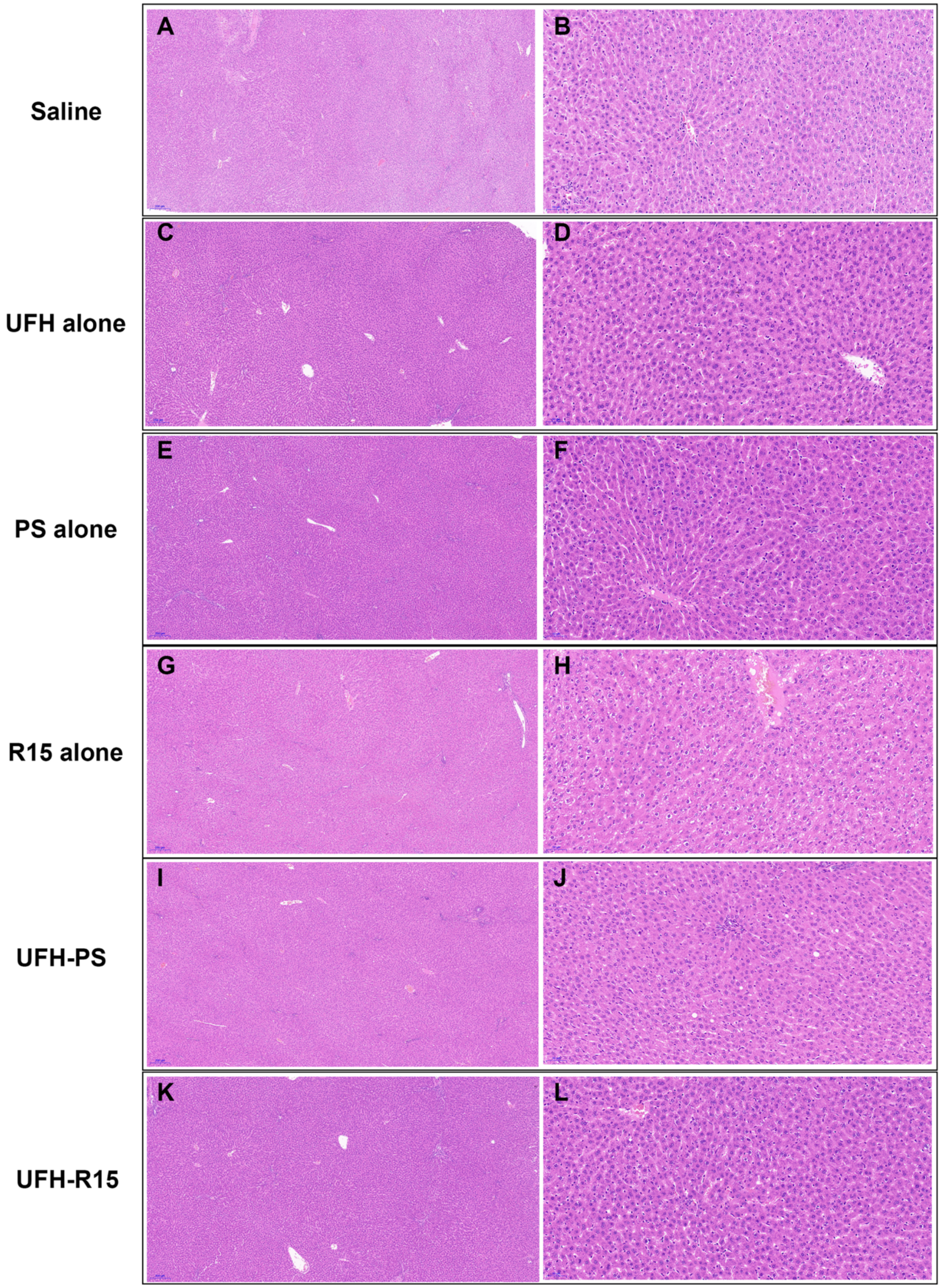

Saline
UFH alone
PS alone
R15 alone
UFH-PS
UFH-R15
A
B
C
D
E
F
G
H
I
J
K
L

Figure S6. Microscopic observation of liver from Wistar rats at 1 h after drug administration. Photomicrographs of histological sections of liver were from Wistar rats at 1 h after treatment with saline (A and B), UFH 900 $U{\cdot}kg^{-1}$ (C and D), PS 900 $U{\cdot}kg^{-1}$ (E and F), R15 900 $U{\cdot}kg^{-1}$ (G and H), UFH 900 $U{\cdot}kg^{-1}$ + PS 900 $U{\cdot}kg^{-1}$ (I and J), and UFH 900 $U{\cdot}kg^{-1}$ + R15 900 $U{\cdot}kg^{-1}$ (K and L). Saline-treated rats were taken as a control group. Images (A, C, E, G, I and K) were taken using the 5× magnification and images (B, D, F, H, J, and L) were taken using 20× magnification. The scale bar is 200 µm (left column) and 50 µm (right column), respectively.

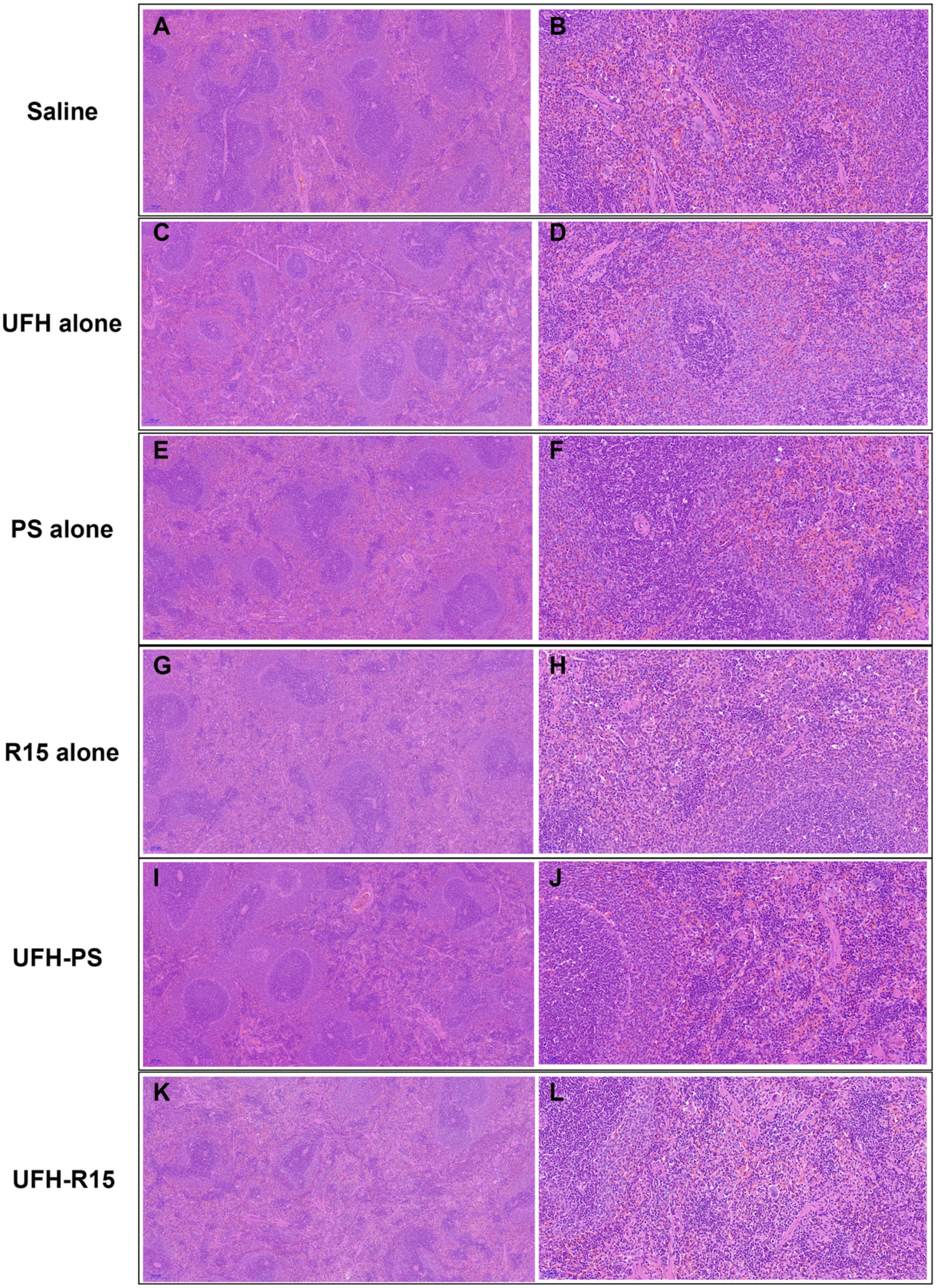
Saline
UFH alone
PS alone
R15 alone
UFH-PS
UFH-R15
A
B
C
D
E
F
G
H
I
J
K
L

Figure S7. Microscopic observation of spleen from Wistar rats at 1 h after drug administration. Photomicrographs of histological sections of spleen were from Wistar rats at 1 h after treatment with saline (A and B), UFH 900 U·kg^{-1} (C and D), PS 900 U·kg^{-1} (E and F), R15 900 U·kg^{-1} (G and H), UFH 900 U·kg^{-1} + PS 900 U·kg^{-1} (I and J), and UFH 900 U·kg^{-1} + R15 900 U·kg^{-1} (K and L). Saline-treated rats were taken as a control group. Images (A, C, E, G, I and K) were taken using the 5× magnification and images (B, D, F, H, J, and L) were taken using 20× magnification. The scale bar is 200 µm (left column) and 50 µm (right column), respectively.

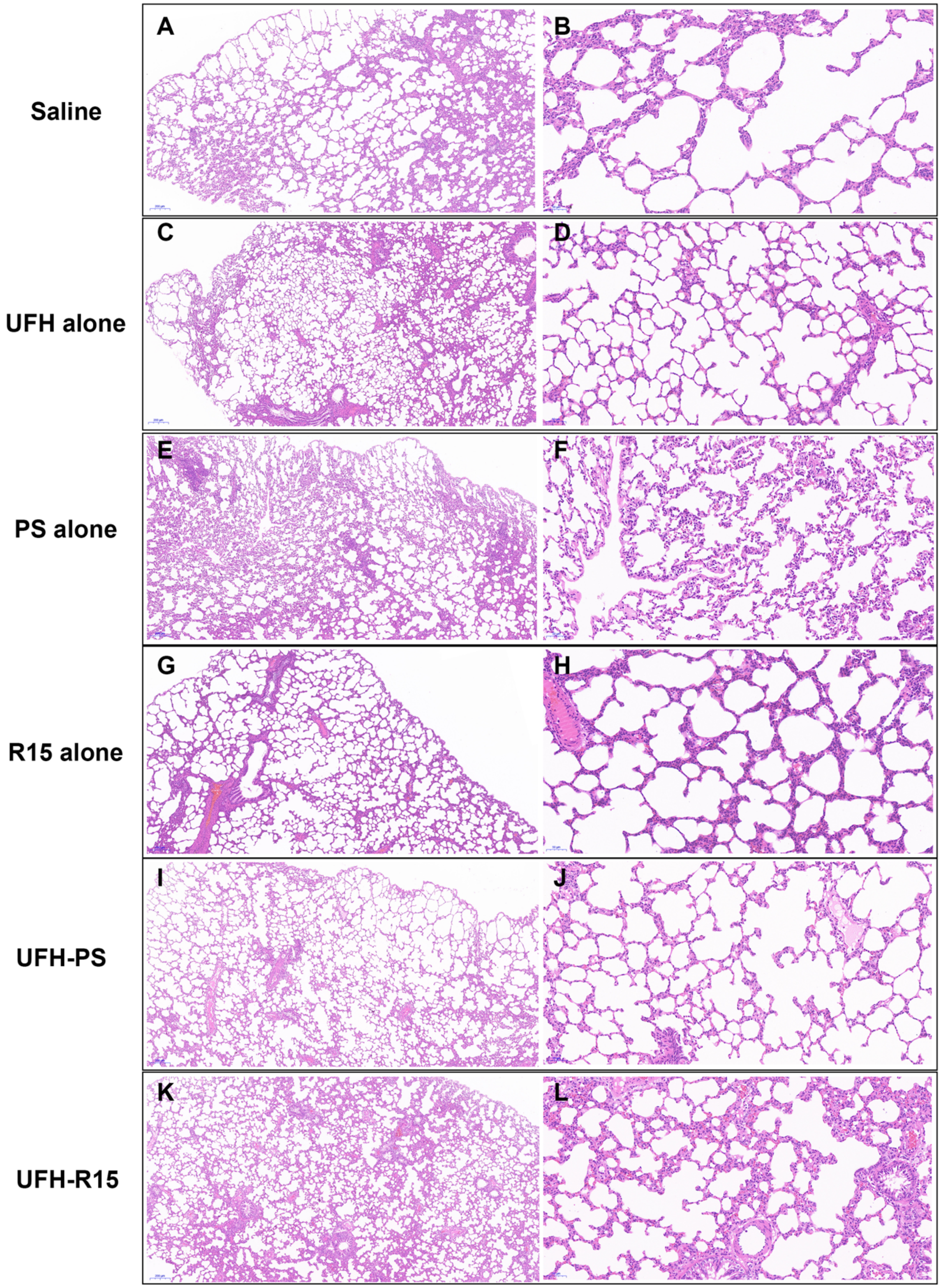
A
B
Saline
C
D
UFH alone
E
F
PS alone
G
H
R15 alone
I
J
UFH-PS
K
L
UFH-R15

Figure S8. Microscopic observation of lung from Wistar rats at 1 h after drug administration. Photomicrographs of histological sections of lung were from Wistar rats at 1 h after treatment with saline (A and B), UFH 900 U·kg^{-1} (C and D), PS 900 U·kg^{-1} (E and F), R15 900 U·kg^{-1} (G and H), UFH 900 U·kg^{-1} + PS 900 U·kg^{-1} (I and J), and UFH 900 U·kg^{-1} + R15 900 U·kg^{-1} (K and L). Saline-treated rats were taken as a control group. Images (A, C, E, G, I and K) were taken using the 5× magnification and images (B, D, F, H, J, and L) were taken using 20× magnification. The scale bar is 200 µm (left column) and 50 µm (right column), respectively.

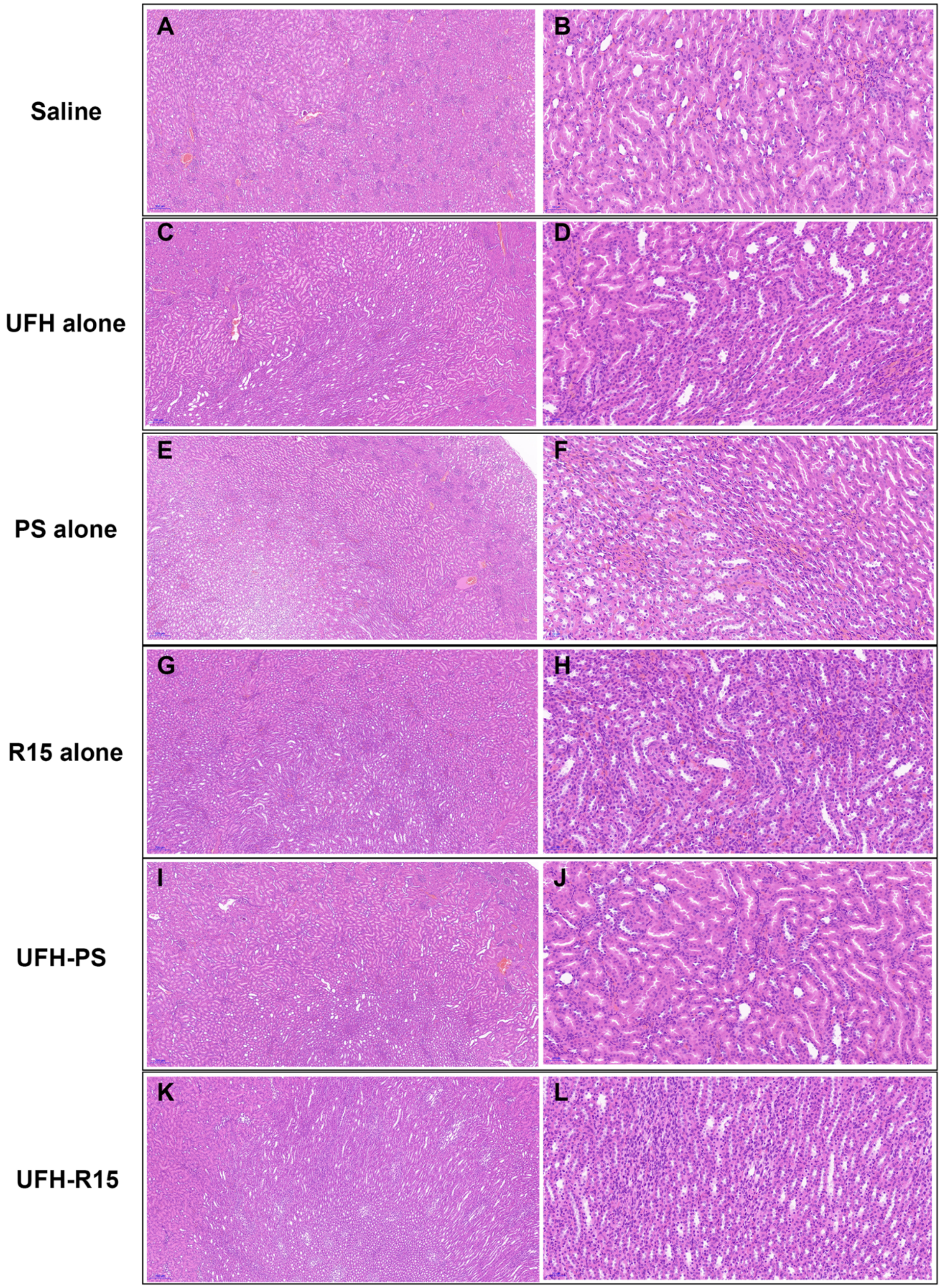
Saline
UFH alone
PS alone
R15 alone
UFH-PS
UFH-R15
A
B
C
D
E
F
G
H
I
J
K
L

Figure S9. Microscopic observation of kidney from Wistar rats at 1 h after drug administration. Photomicrographs of histological sections of kidney were from Wistar rats at 1 h after treatment with saline (A and B), UFH 900 U·kg^{-1} (C and D), PS 900 U·kg^{-1} (E and F), R15 900 U·kg^{-1} (G and H), UFH 900 U·kg^{-1} + PS 900 U·kg^{-1} (I and J), and UFH 900 U·kg^{-1} + R15 900 U·kg^{-1} (K and L). Saline-treated rats were taken as a control group. Images (A, C, E, G, I and K) were taken using the 5× magnification and images (B, D, F, H, J, and L) were taken using 20× magnification. The scale bar is 200 µm (left column) and 50 µm (right column), respectively.

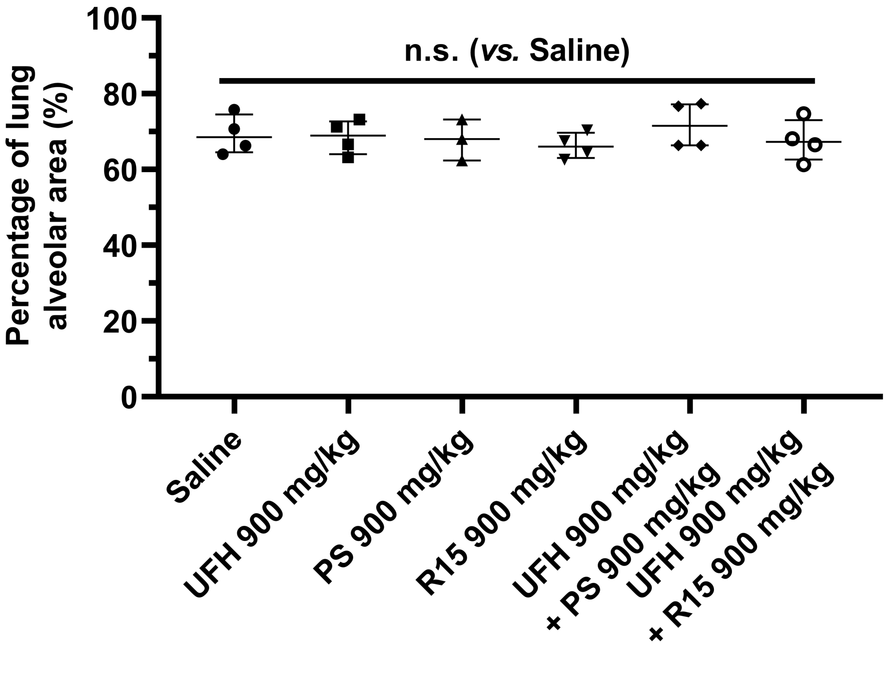

Figure S10. Percentage of lung alveolar areas of Wistar rats. Lungs were harvested at 1 h after drug administration and processed for H&E staining. Color images at 10× magnification were captured and percentage of lung alveolar areas were measured from the 144 images of lungs from 24 rats (4 rats per treatment group). Generally, major airways and blood vessels were avoided from images to measure alveolar areas. For alveolar area measurements, images were

thresholded and analyzed with ImageJ. Histological sections of lung from one rat treated with PS (900 U·kg^{-1}) were damaged during H&E staining, therefore 3 rats were used for calculation of lung alveolar areas. The data are expressed as the median with interquartile range, analyzed with Kruskal–Wallis test followed by Dunnett's multiple comparisons test. n.s. represents $p > 0.05$.

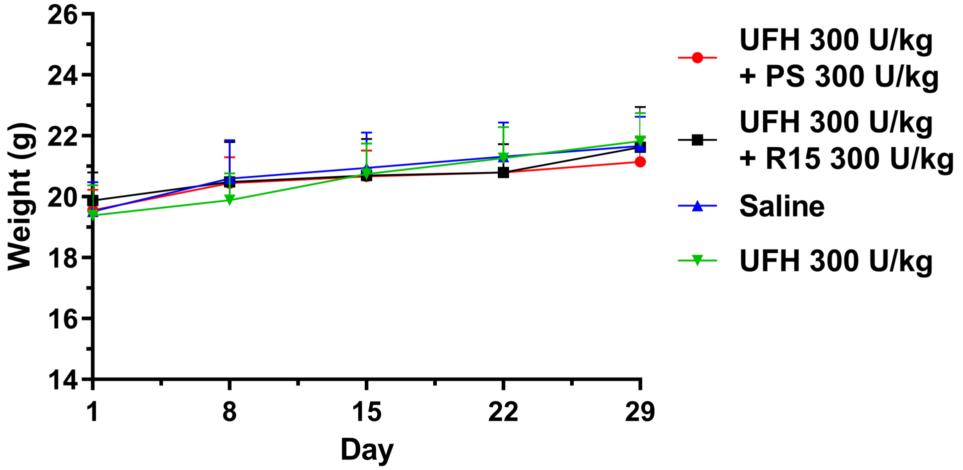

Figure S11. Mean body weight of Balb/c mice (n=16 mice per group) from baseline to day 29. Results are shown as the mean ± SD.

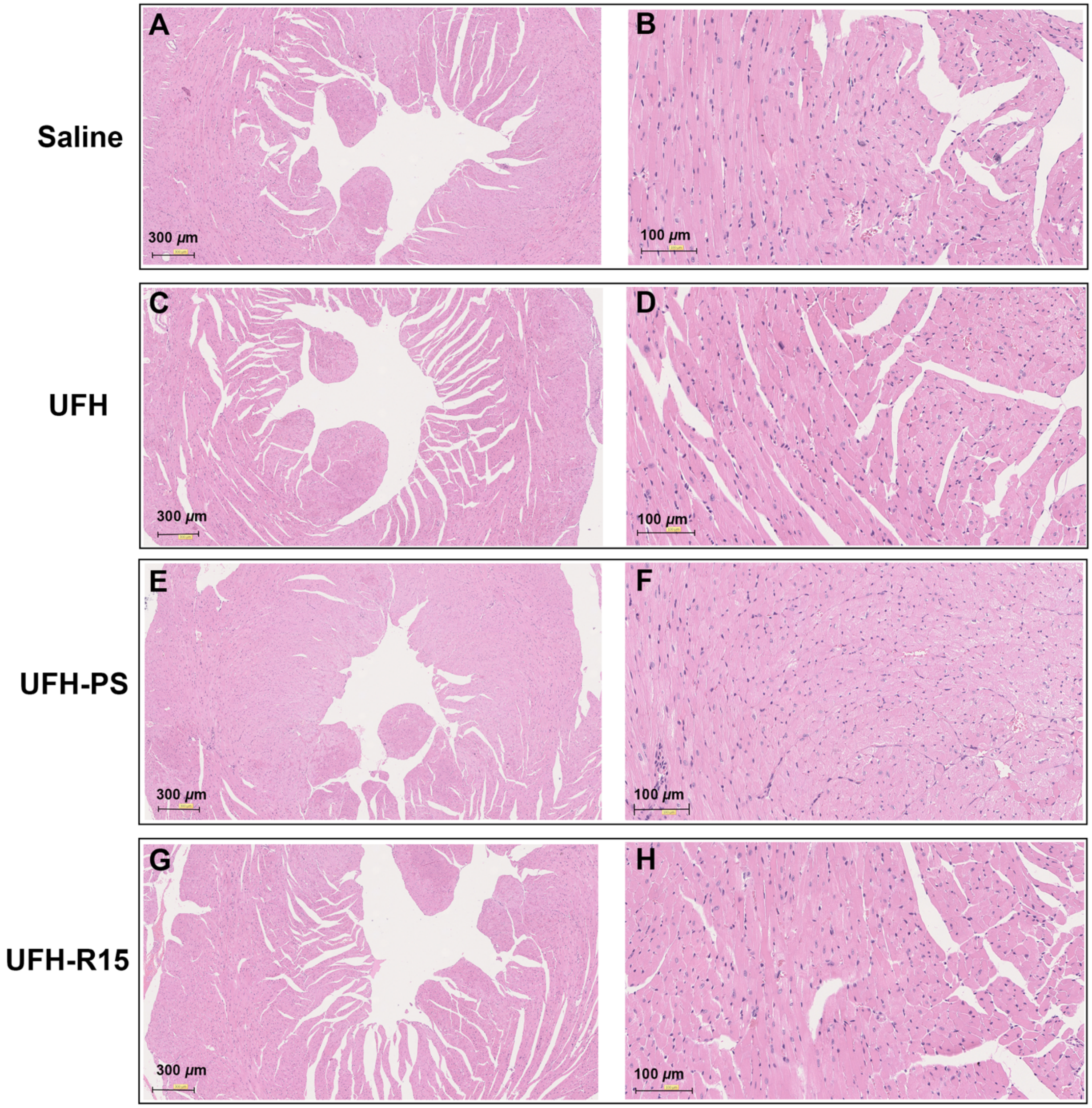

Figure S12. Microscopic observation of heart from Balb/c mice at 6th week. Test substances were given to Balb/c mice once a week for five weeks, and the heart were harvested at 6th week. Saline-treated mice were taken as a control group. Photomicrographs of histological sections of heart were from Balb/c mice with saline (A and B), UFH 300 U·kg^{-1} (C and D), UFH 300 U·kg^{-1} + PS 300 U·kg^{-1} (E and F), and UFH 300 U·kg^{-1} + R15 300 U·kg^{-1} (G and H). Images (A, C, E, and G) were taken

using the 5× magnification and images (B, D, F, and H) were taken using 20× magnification. The scale bar is 300 µm (left column) and 100 µm (right column), respectively.

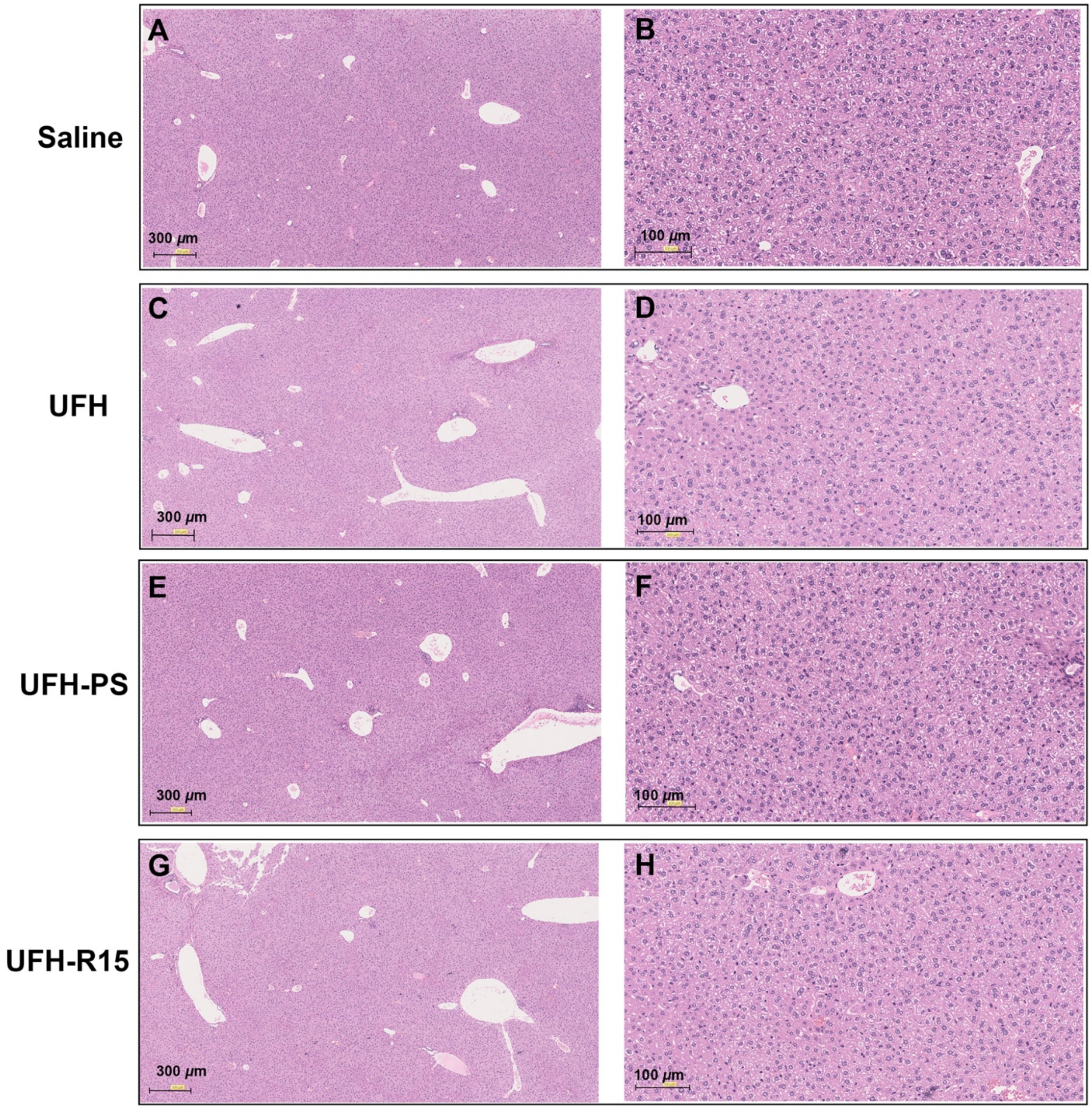

Figure S13. Microscopic observation of liver from Balb/c mice at 6th week. Test substances were given to Balb/c mice once a week for five weeks, and the liver were harvested at 6th week. Saline-

treated mice were taken as a control group. Photomicrographs of histological sections of liver were from Balb/c mice with saline (A and B), UFH 300 $U \cdot kg^{-1}$ (C and D), UFH 300 $U \cdot kg^{-1}$ + PS 300 $U \cdot kg^{-1}$ (E and F), and UFH 300 $U \cdot kg^{-1}$ + R15 300 $U \cdot kg^{-1}$ (G and H). Images (A, C, E, and G) were taken using the 5× magnification and images (B, D, F, and H) were taken using 20× magnification. The scale bar is 300 µm (left column) and 100 µm (right column), respectively.

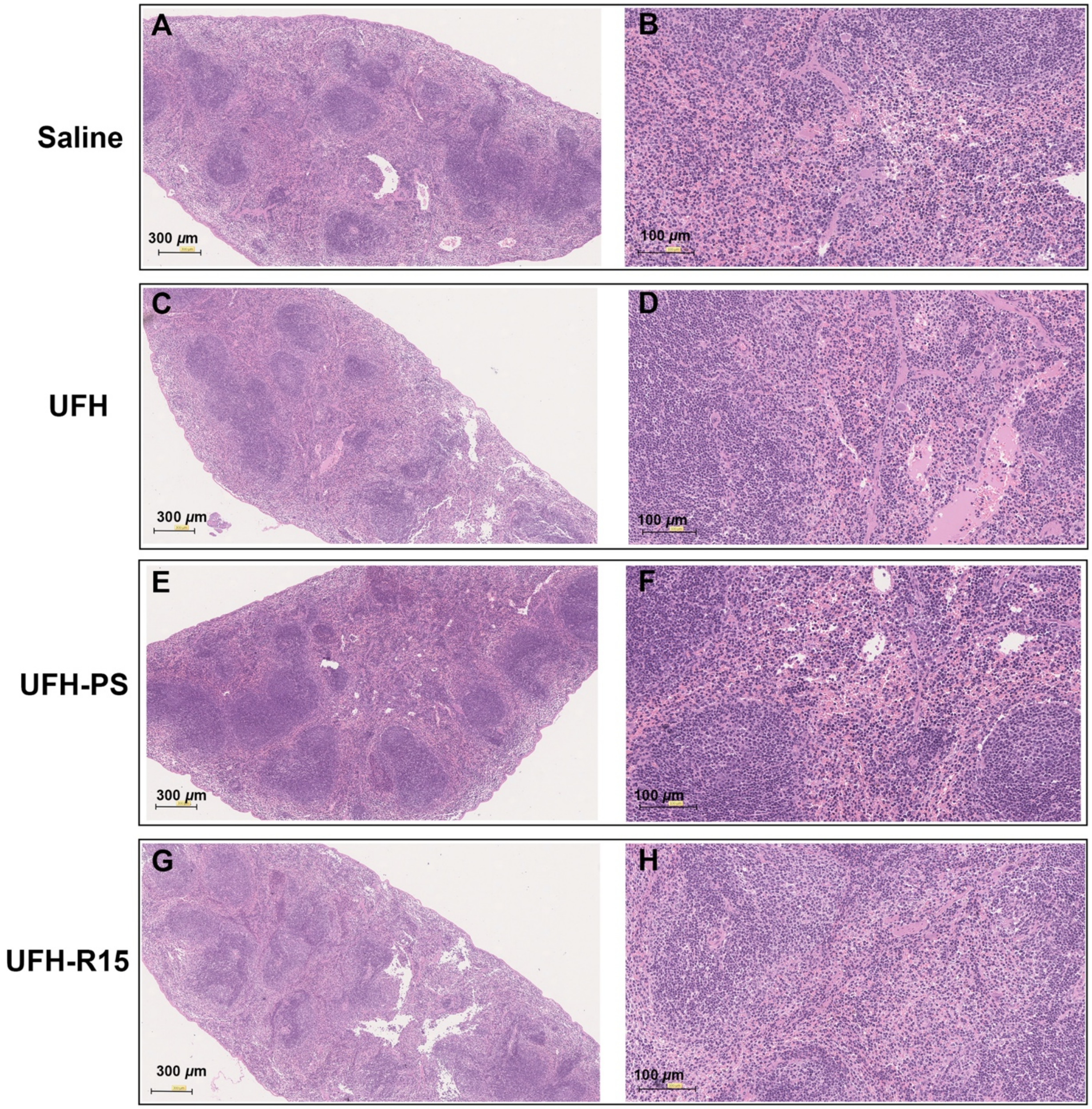

Figure S14. Microscopic observation of spleen from Balb/c mice at 6th week. Test substances were given to Balb/c mice once a week for five weeks, and the spleen were harvested at 6th week. Saline-treated mice were taken as a control group. Photomicrographs of histological sections of spleen were from Balb/c mice with saline (A and B), UFH 300 U·kg^{-1} (C and D), UFH 300 U·kg^{-1} + PS 300 U·kg^{-1} (E and F), and UFH 300 U·kg^{-1} + R15 300 U·kg^{-1} (G and H). Images (A, C, E, and G)

were taken using the 5× magnification and images (B, D, F, and H) were taken using 20× magnification. The scale bar is 300 μm (left column) and 100 μm (right column), respectively.

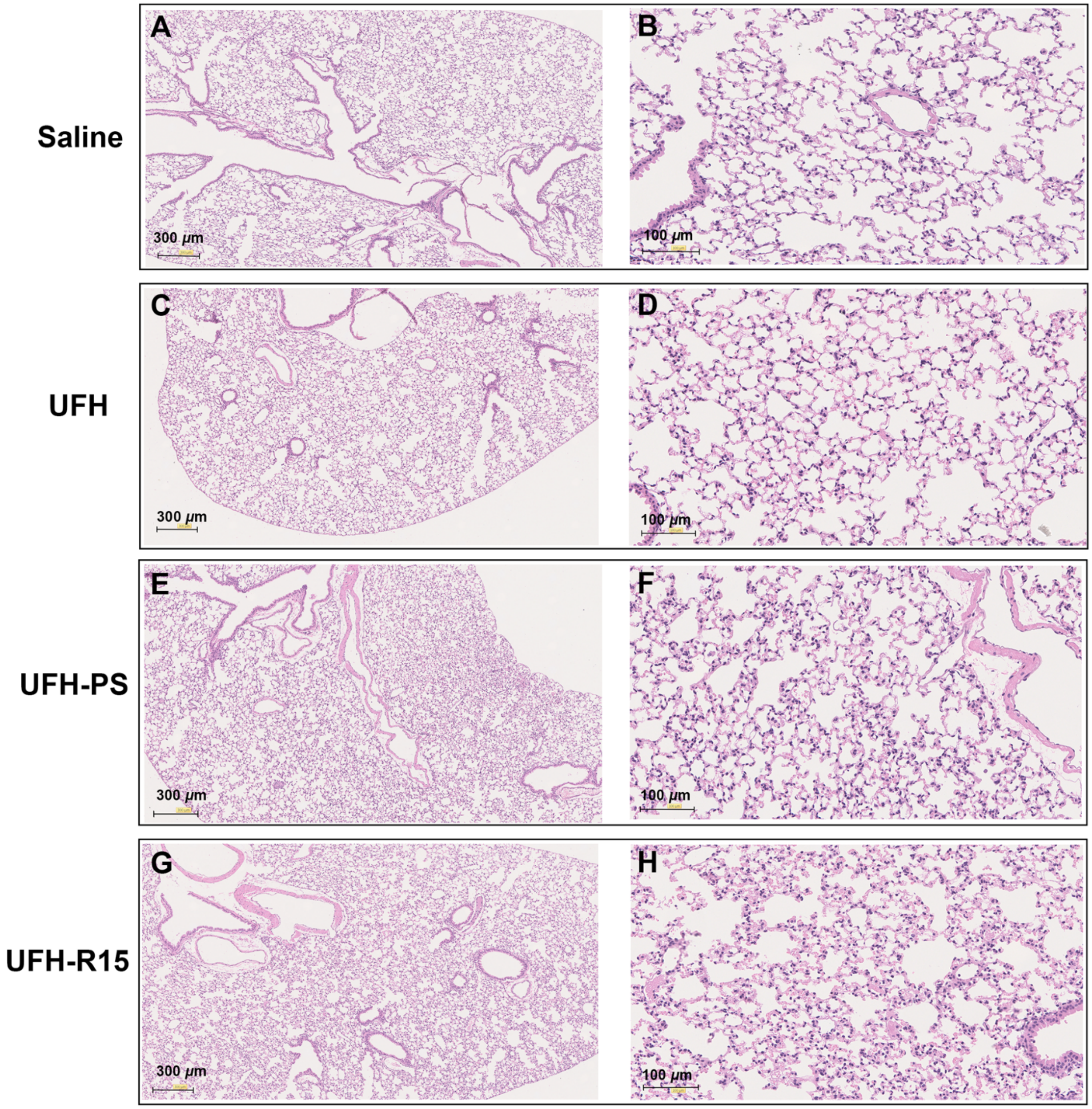

Figure S15. Microscopic observation of lung from Balb/c mice at 6th week. Test substances were given to Balb/c mice once a week for five weeks, and the lung were harvested at 6th week. Saline-

treated mice were taken as a control group. Photomicrographs of histological sections of lung were from Balb/c mice with saline (A and B), UFH 300 U·kg^{-1} (C and D), UFH 300 U·kg^{-1} + PS 300 U·kg^{-1} (E and F), and UFH 300 U·kg^{-1} + R15 300 U·kg^{-1} (G and H). Images (A, C, E, and G) were taken using the 5× magnification and images (B, D, F, and H) were taken using 20× magnification. The scale bar is 300 μm (left column) and 100 μm (right column), respectively.

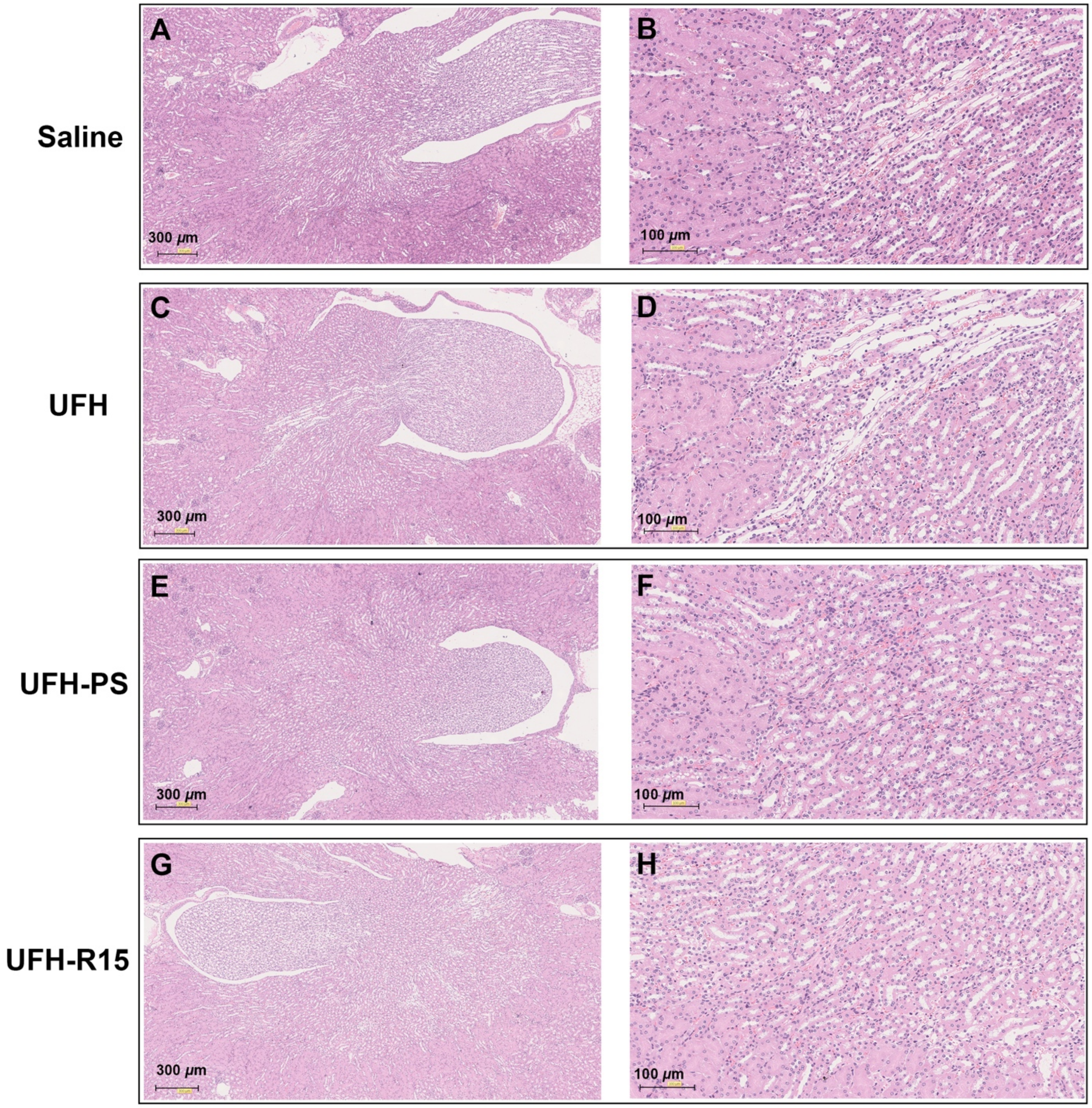

Figure S16. Microscopic observation of kidney from Balb/c mice at 6th week. Test substances were given to Balb/c mice once a week for five weeks, and the kidney were harvested at 6th week. Saline-treated mice were taken as a control group. Photomicrographs of histological sections of kidney were from Balb/c mice with saline (A and B), UFH 300 U·kg^{-1} (C and D), UFH 300 U·kg^{-1} + PS 300 U·kg^{-1} (E and F), and UFH 300 U·kg^{-1} + R15 300 U·kg^{-1} (G and H). Images (A, C, E, and G)

were taken using the 5× magnification and images (B, D, F, and H) were taken using 20× magnification. The scale bar is 300 µm (left column) and 100 µm (right column), respectively.

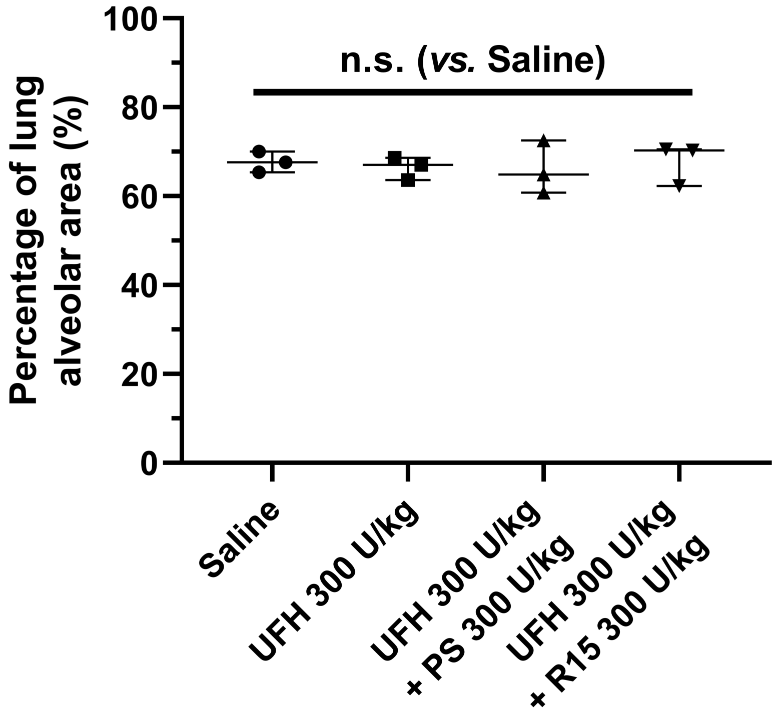

Figure S17. Percentage of lung alveolar areas of Balb/c mice. Lungs were harvested at 6th week and processed for H&E staining. Color images at 13.5× magnification were captured and percentage of lung alveolar areas were measured from the 72 images of lungs from 12 mice (3 mice per treatment group). Generally, major airways and blood vessels were avoided from images to measure alveolar areas. For alveolar area measurements, images were thresholded and analyzed with ImageJ. The data are expressed as the median with interquartile range, analyzed by Kruskal–Wallis test followed by Dunnett's multiple comparisons test. n.s. represents $p > 0.05$.

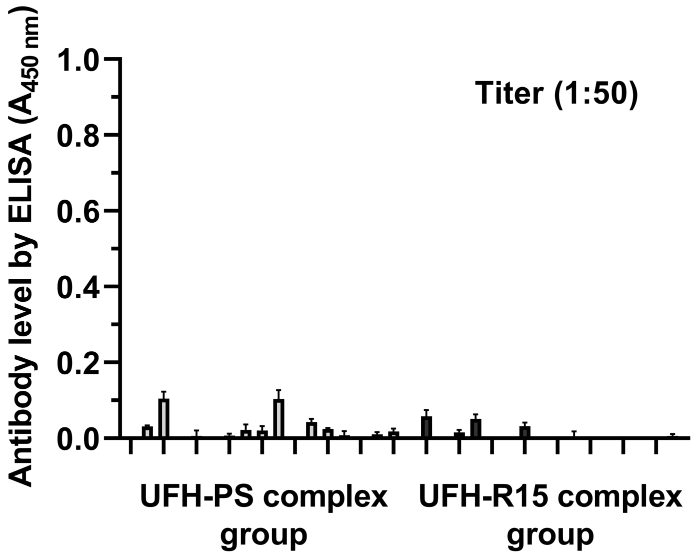

Figure S18. Detection of UFH-PS complex and UFH-R15 complex antibodies by ELISA assays. Heparinized Balb/c mice (n=16) were reversal with UFH-PS complex or UFH-R15 complex once a week for five weeks. High-affinity microplates coated with UFH-PS complex and UFH-R15 complex were used to detect antibody levels. Sera were diluted 50 times with PBS buffer for detection. The samples were measured by an ELISA method in triplicate.

Table S1. Vital organ index of Wistar rats treated with test substances (n=4 rats per group)

Saline-treated rats were taken as a control group. Organ index were calculated from ratio of organ weight to body weight (g/g) *100 to reveal potential organ damages. Data are shown as the mean ± SD, analyzed with a one-way ANOVA followed by Dunnett's multiple comparisons test *(vs.* saline group). No statistical significance was found in each group.

Organ	Organ weight / body weight (g/g) * 100					
	Saline	UFH 900 U·kg^{-1}	PS 900 U·kg^{-1}	R15 900 U·kg^{-1}	UFH 900 U·kg^{-1} + PS 900 U·kg^{-1}	UFH 900 U·kg^{-1} + R15 900 U·kg^{-1}
Heart	0.31±0.02	0.33±0.03	0.33±0.03	0.34±0.03	0.31±0.02	0.34±0.03

Liver	4.03±0.26	3.95±0.32	3.95±0.28	3.83±0.46	3.89±0.45	3.91±0.12
Spleen	0.32±0.13	0.28±0.02	0.37±0.03	0.38±0.15	0.33±0.11	0.35±0.10
Lung	0.56±0.04	0.60±0.18	0.64±0.08	0.56±0.02	0.58±0.11	0.55±0.05
Kidney	0.79±0.05	0.84±0.11	0.82±0.06	0.81±0.07	0.81±0.10	0.84±0.08
